# Ratiometric signaling produces robust temporal integration for accurate cellular gradient sensing

**DOI:** 10.1101/2025.04.18.649595

**Published:** 2026-02-18

**Authors:** Debraj Ghose, James Nolen, Kaiyun Guan, Timothy C. Elston, Daniel J. Lew

**Affiliations:** Wyss Institute for Biologically Inspired Engineering, Harvard University, Boston, MA, 02215, USA; Department of Mathematics, Duke University, Durham, NC, 27708, USA; Curriculum in Bioinformatics and Computational Biology, University of North Carolina at Chapel Hill, Chapel Hill, NC, 27599, USA; Department of Pharmacology and Computational Medicine Program, University of North Carolina at Chapel Hill, Chapel Hill, NC, 27599, USA; Department of Biology, Massachusetts Institute of Technology, Cambridge, MA, 02139, USA

## Abstract

Cells excel at interpreting noisy chemical gradients to guide fertilization, development, and immune responses, but the mechanisms underlying this remarkable ability remain poorly understood. Previous work showed that some G protein signaling pathways can overcome challenges from uneven receptor distribution by using a ratiometric signaling strategy. In this mechanism, G proteins receive information from both bound and unbound receptors, unlike classical signaling where only bound receptors contribute. Here, we show that ratiometric signaling also provides an unexpected ability to suppress noise from low receptor numbers. The benefit stems from each G protein remembering the last receptor state it encountered, so that at any instant, ratiometric G protein collectives reflect time-averaged receptor activity. Unlike classical signaling, this averaging remains unbiased and accurate across the varying ligand concentrations present in a spatial gradient. Using theory and simulations, we demonstrate that this averaging mechanism allows cells to surpass theoretical limits for gradient detection from instantaneous receptor information alone. Our findings reveal how ratiometric biochemical architectures enable robust temporal integration across spatially varying signals, providing cells with enhanced directional accuracy under noisy conditions.

## 1. INTRODUCTION

Cells are exceptional at extracting directional information from their chemical environment, often under complex and noisy conditions that demand high accuracy. Some of the earliest direct experimental evidence that cells respond to chemical gradients emerged in the late 19th century, with studies involving bacteria clustering near oxygen-rich zones [1, 2] and plant spermatozoids finding female reproductive organs [3]. Since then, chemotactic or chemotropic responses by cells or subcellular components have been documented in pollen tube guidance [4, 5], protozoa [6–8], fungi [9–14], immune cells [15, 16], animal sperm [17–19], neurons [20, 21], developmental processes [22], cancer cells [23–25], and many other systems. Together, these observations broadly illustrate the diverse scenarios under which gradient-driven cell guidance occurs.

In all cases, cells detect chemical signals through binding to cell-surface receptors that engage internal signaling pathways [26, 27]. Using these receptors, a cell can in principle estimate the direction of a ligand gradient by measuring the difference in chemical concentration across its width. However, the rapid movement of fast-swimming cells like bacteria or sperm causes them to collide with ligand molecules more often at their front than at their back. This differential collision rate can make the concentration gradients they perceive across their width appear hundreds of times steeper than the actual environmental gradients [28]. Such cells have developed “temporal sensing” strategies that monitor concentration changes over time as they move through space. In contrast, a majority of larger eukaryotic cells, which are slower moving or non-motile, employ a “spatial sensing” strategy that compares concentrations of chemicals on different sides of the cell to infer gradient direction. Once a direction is established, eukaryotic cells can polarize and move or grow toward (or away from) higher ligand concentrations. Even so, these spatial measurements can be limited by gradient measurements with low signal to noise, especially for smaller cells. These limits have been theoretically studied for both absolute ligand concentration measurement [28–30] and gradient measurement [31, 32], grounded in considerations of ligand-binding noise. A well-appreciated strategy to enhance low signal is to use time-averaging of receptor signals, which can significantly improve both concentration measurement and gradient detection. However, molecular mechanisms for averaging receptor signals and improving gradient detection remain poorly understood.

In classical G protein signaling, G proteins are activated by ligand-bound receptors and deactivated at a uniform rate. Here, inspired by the mating response of budding yeast, we examined an alternate strategy: ratiometric signaling, in which G proteins are activated by bound receptors and deactivated by unbound receptors [33, 34]. This architecture means that downstream signaling depends on the ratio of active to inactive receptor levels rather than absolute number of active receptors.

We discovered that ratiometric signaling enables robust time averaging that significantly outperforms classical signaling. This mechanism arises from collectives of downstream G proteins that remember past receptor states. While classical signaling fails to maintain accurate averaging across the varying concentrations present in a spatial gradient, ratiometric signaling automatically adjusts to different concentrations. Furthermore, this mechanism allows cells to surpass theoretical limits for instantaneous gradient sensing, revealing how cells achieve their remarkable ability to interpret noisy directional signals. These results reveal design principles underlying robust spatial gradient sensing and suggest strategies for engineering synthetic systems that can navigate complex chemical environments.

## II. RESULTS

### A. Instantaneous limits for gradient estimation by receptors

**FIG. 1.**
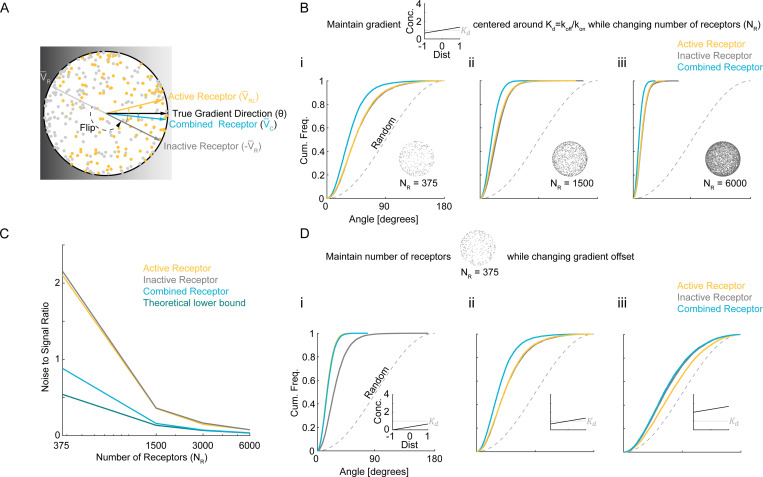
Estimating gradient direction using information from both active and inactive receptors. (**A**) Schematic depicting a spherical cell with receptors randomly distributed on its surface. Each receptor can be active (ligand-bound, yellow) or inactive (unbound, gray), and the gradient direction is inferred by summing receptor vectors and normalizing the sums to obtain unit vectors ${\bar{V}}_{RL}$, ${\bar{V}}_{R}$, and ${\bar{V}}_{C}$. Note that ${\bar{V}}_{R}$ is flipped to obtain $-{\bar{V}}_{R}$ to estimate gradient direction. (**B**) Cumulative distribution functions (CDFs) of angular error for the estimates ${\bar{V}}_{RL}$, $-{\bar{V}}_{R}$, ${\bar{V}}_{C}$, for three different receptor numbers $\left(N_{R}=375,1500,6000\right)$, where $K_{d}=6nM$ and the gradient is centered around $C_{0}=K_{d}$ and ranges from $0.66\;K_{d}$ to $1.33\;K_{d}$ (i.e., b/L=0.33). The dashed curve indicates the angular error for an estimate chosen uniformly at random over the cell. (**C**) Noise-to-signal ratio (NSR) comparing active-receptor sensing (yellow), inactive-receptor sensing (gray), and the combined-receptor estimate (blue) to the theoretical lower bound (dark green). Higher receptor counts reduce NSR, with the combined-receptor approach consistently outperforming either active- or inactive-only sensing. (**D**) Cumulative distribution functions (CDFs) of the angular error for the estimates ${\bar{V}}_{RL}$, $-{\bar{V}}_{R}$, ${\bar{V}}_{C}$ at fixed $N_{R}=375$ but varying the gradient’s offset relative to $K_{d}:C_{0}/K_{d}\in \{1/3,1,3\}$. As the mean concentration shifts, active and inactive receptors each capture different portions of the ligand profile, and combining their information again improves direction inference.

To evaluate the potential benefits or pitfalls of a ratiometric sensing strategy, we need a metric that tells us how accurately a theoretical cell can infer the direction of a ligand gradient. Previous studies have compared the ligand concentrations sensed by the front and back halves of a cell, thereby determining whether the difference (signal) exceeds the noise [28, 31]. However, splitting the cell in half presupposes prior knowledge of the axis along which concentration changes occur. Because a real cell generally does not have that information, other work has instead estimated the cell’s “best guess” of the gradient direction by summing the vectors from the cell center to all active receptors [32, 35] (Fig. 1A).

The resulting active receptor vector, $V_{RL}$, provides an estimate of the gradient direction pointing towards the highest ligand concentration. Comparing $V_{RL}$ with the actual gradient direction provides an angular measure of how closely the cell’s guess matches reality. In principle, one could also compute the gradient direction from inactive receptors alone to produce a “best guess” vector $V_{R}$ pointing towards the lowest ligand concentration. To combine information from both active and inactive receptors, we can calculate $V_{C}=V_{RL}-V_{R}$ (Fig. 1A).

In what follows, we simulate a spherical cell in three dimensions, with $N_{R}$ receptors randomly distributed over its surface. As receptor diffusion on the membrane is slow, especially in budding yeast $\left(∼0.0005\mu m^{2}/s\right)$ [33, 36], we neglect it. Receptors can switch between active and inactive states at rates that depend on local ligand concentration C(x); in statistical equilibrium, the probability of a receptor being active at a given time is

(1) $$\mathbb{P}(\text{receptor at}\;x\;\text{is active})=\frac{C(x)}{C(x)+K_{d}}.$$

We expose the spherical cell to linear ligand gradients of the form

(2) $$C(x,\theta )=C_{0}(1+b(x\cdot \theta )/L),$$

where θ is the direction vector, $C_{0}$ is the mid-point (average) ligand concentration, and x is a point on the sphere of radius L centered at the origin. We first assume the gradient is centered around $K_{d}$ (i.e., $C_{0}=K_{d}$). Molecular noise from ligand binding/unbinding causes estimates of the gradient direction θ to fluctuate over time. We quantify directional accuracy by examining the empirical cumulative distribution functions for the angular deviation of ${\bar{V}}_{RL}$, $-{\bar{V}}_{R}$, and ${\bar{V}}_{C}$ (the normalized summed vectors) relative to θ across many independent realizations (Fig. 1 B i-iii).

As ${\bar{V}}_{c}$, ${\bar{V}}_{RL}$, and $-{\bar{V}}_{R}$ are each statistical estimators of gradient direction, we can compare them to the Cramér-Rao bound [37], which provides a rigorous lower bound on the variance of $\bar{V}$ from the true direction θ, relative to the signal strength $\left\|E[\bar{V}]\right\|$. Accuracy of the direction estimate is quantified by a “noise-to-signal ratio” (NSR), and the statistical bound takes the form

(3) $$NSR\geq \mathrm{trace}\left(I_{R}^{-1}\right)$$

where $I_{R}$ is a Fisher information matrix that can be computed numerically. In the weak-signal regime (small slope, b) this bound is approximated by

(4) $$NSR\geq \mathrm{trace}\left(I_{R}^{-1}\right)\approx \frac{6}{N_{R}b^{2}}\frac{{\left(C_{0}+K_{d}\right)}^{2}}{K_{d}C_{0}},$$

which shows that the minimal achievable variance in the estimated direction decreases proportionally to $1/N_{R}$ and depends inversely on $b^{2}$, thereby highlighting the advantage of stronger gradients and higher receptor number (details of this bound and a derivation of (4) appear in the Supplementary Information). A related bound is derived in [32] for a different model.

A comparison with this theoretical lower bound (Equation 3) shows that the estimates ${\bar{V}}_{c}$, ${\bar{V}}_{RL}$, and $-{\bar{V}}_{R}$ are indeed noisy, leaving ample room for improvement, especially at low $N_{R}$ (Fig. 1 C). At high $N_{R}$, the variance of ${\bar{V}}_{c}$, $-{\bar{V}}_{R}$, and ${\bar{V}}_{RL}$ decrease along with the Cramér-Rao bound, which scales as $1/N_{R}$ (Fig. 1C). For $C_{0}=K_{d}$, the variance of the combined estimate ${\bar{V}}_{C}$ is roughly half that of either ${\bar{V}}_{RL}$ or ${\bar{V}}_{R}$, as expected from the $1/N_{R}$ scaling of the optimal variance and the fact that roughly half of the receptors are active in this regime. Effectively, ${\bar{V}}_{C}$ draws on twice the data that either ${\bar{V}}_{RL}$ or $-{\bar{V}}_{R}$ alone can provide, demonstrating that incorporating both ligand-bound and unbound receptors significantly enhances directional sensing.

To determine whether these conclusions extend to other gradients, we simulated a range of linear gradients covering different mean ligand concentrations (Fig. 1D). As the ligand concentration rises, saturation becomes more pronounced, so a larger fraction of receptors are active and the signal is diminished. Consistent with that expectation, $V_{RL}$ performs increasingly poorly at higher concentrations (Figure 1D i-iii). Though $-{\bar{V}}_{R}$ also degrades at high concentrations, it does so differently from $V_{RL}$. When the gradient is centered below $K_{d}$, $V_{RL}$ outperforms $-V_{R}$ (Fig. 1D i); at $K_{d}$, $V_{RL}$ and $-V_{R}$ perform similarly (Fig. 1D iii); above $K_{d}$, $-V_{R}$ becomes superior (Fig. 1D iii). This pattern suggests that at low ligand concentrations, the scarce active receptors provide more information than the many inactive ones, whereas at high ligand concentrations, the fewer inactive receptors carry more information. The combined vector $V_{C}$ performs as well as or better than either $V_{RL}$ or $-V_{R}$ across the same tested range (Fig. 1 D i-iii). Hence, pooling signals from both active and inactive receptors broadens the range of gradients that can be decoded accurately.

### B. Ratiometric signaling biochemistry greatly enhances gradient estimation

Until now, we have imagined that a cell could apply some procedure to calculate $V_{C}$ (and extract the direction ${\bar{V}}_{C}$), but we neglected the actual biochemical processes by which such calculations necessarily arise. Indeed, any downstream signaling step cannot preserve 100% of the information available at an earlier step, as noise in the interpretation mechanism will inevitably degrade the signal. For the ubiquitous and large family of G protein coupled receptors (GPCRs) that signal to downstream heterotrimeric G proteins, the biochemical mechanisms have been elucidated, and we consider here a simplified scheme in which a cell contains $N_{G}$ G proteins that diffuse on the 2D surface of the spherical cell and can become activated or inactivated. Activation occurs with some probability upon collision between a diffusing inactive G protein and a ligand-bound receptor.

**FIG. 2.**
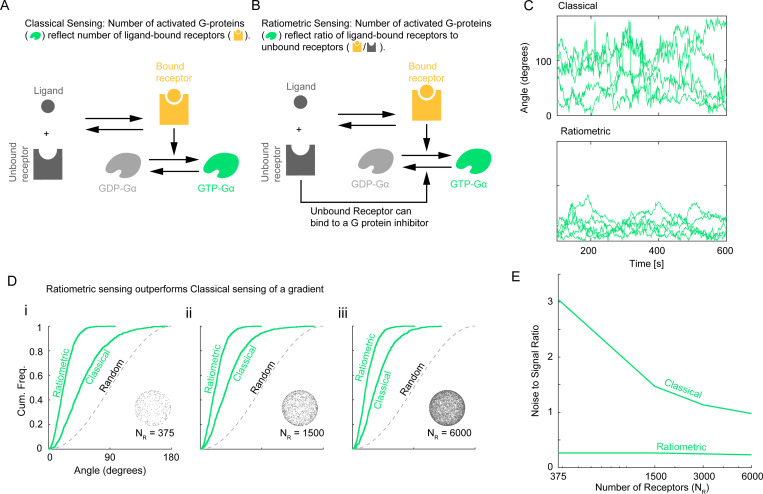
Ratiometric gradient sensing outperforms classical gradient sensing across a range of receptor numbers $N_{R}$. (**A**) Schematic of reactions that constitute classical sensing. (**B**) Schematic of reactions that constitute ratiometric sensing. (**C**) 5 sample traces each of direction derived from G protein distribution over time for ratiometric and classical sensing strategies. $N_{R}=375$, $N_{G}=2500$, $D_{G}=0.002\mu m^{2}s^{-1}$, $k_{on}=0.0167s^{-1}nM^{-1}$, $k_{off}=0.1s^{-1}$, $K_{d}=6nM$. (**D**) Cumulative distribution functions of angular error comparing gradient sensing between ratiometric $\left({\bar{V}}_{G}^{r}\right)$ and classical $\left({\bar{V}}_{G}^{c}\right)$ scenarios. $N_{R}=[375,3000,6000]$ using the parameters in (C). (**E**) Noise to signal ratio of direction derived from G protein distribution compared between classical and ratiometric sensing strategies. $N_{R}=[375,1500,3000,6000]$ using the parameters in (C). For classical sensing, the G protein off-rate was assigned according to $N_{R}\mapsto k_{go}$ as $[375,1500,3000,6000]\mapsto [0.0083,0.0357,0.0769,0.1687]{\text{s}}^{-1}$.

Following [33], we explore two different models of G protein inactivation (Fig. 2). In the *classical* model, inactivation occurs at a spatially uniform rate, $k_{i}^{\prime }$, that is independent of receptor occupancy (Fig. 2A). In contrast, in the *ratiometric* model, inactivation occurs with some probability upon collision between a diffusing active G protein and an unbound receptor (Fig. 2B). We adjust the uniform inactivation rate of the classical model so that matched classical and ratiometric simulations have the same steady-state abundance of active G proteins, thereby permitting fair comparisons [33]. Note that unlike the ratiometric model, the classical model does not incorporate information about the spatial distribution of inactive receptors.

In a ratiometric system, because ligand binding simultaneously increases the number of active receptors and decreases the number of inactive receptors, these opposing effects can amplify fluctuations due to molecular noise [38]. Indeed, [39] suggests that for a fixed ligand concentration, a cell’s measurement of ligand levels is noisier under a ratiometric (or “concerted”) mechanism than under the classical mechanism. Nevertheless, this need not imply that direction sensing becomes less reliable. For example, suppose $\left(F_{r},B_{r}\right)$ and $\left(F_{c},B_{c}\right)$ are pairs of random variables representing the active G protein concentration near the cell’s front (F) and back (B), under the ratiometric (r) or classical (c) mechanisms. It may be that $CV\left(F_{c}\right)<CV\left(F_{r}\right)$ and $CV\left(B_{c}\right)<CV\left(B_{r}\right)$, yet $F_{r}-B_{r}>F_{c}-B_{c}$ (or $F_{r}/B_{r}>F_{c}/B_{c}$) still holds with high probability. (CV denotes coefficient of variation.) In other words, although the “signal” at any given location may be noisier in the ratiometric mechanism, the overall direction information (e.g., the difference F−B or ratio F/B) can remain more robust compared to the classical system (see Section 5 and Fig. S6–S8 in the Supplement).

To compare direction sensing between classical and ratiometric systems, we calculated resultant vectors for active G proteins in a manner analogous to the previously discussed active receptor vector $V_{RL}$ (Fig. 1A). We denote the resultant vectors of active G proteins under the ratiometric and classical models by $V_{G}^{r}$ and $V_{G}^{c}$, respectively, and their normalized directions by ${\bar{V}}_{G}^{r}$ and ${\bar{V}}_{G}^{c}$. For ligand gradients near the receptor $K_{d}$, the ratiometric model $\left(V_{G}^{r}\right)$ yields a significantly more accurate estimate of the gradient direction than the classical model $\left(V_{G}^{c}\right)$ as demonstrated by temporal traces (Fig. 2C) (Movies S1-S6) and cumulative distribution functions (Fig. 2D) of $V_{G}^{c}$ direction relative to gradient direction. Consequently, ratiometric sensing markedly enhances the accuracy of spatial gradient detection, even though it may sacrifice some accuracy in estimating the overall ligand concentration.

**FIG. 3.**
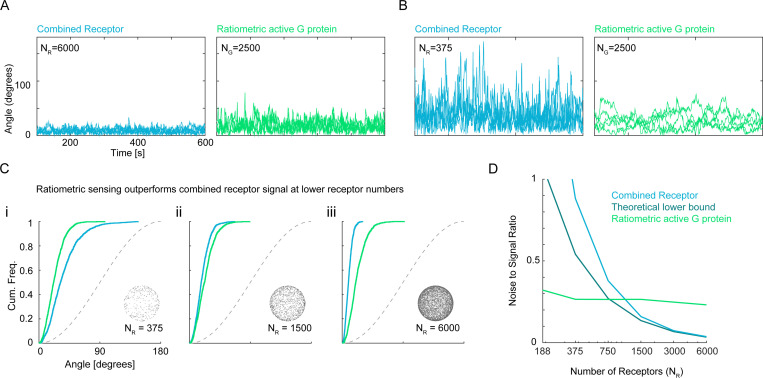
Ratiometric sensing allows G proteins to outperform instantaneous theoretical limit. (**A)** Temporal tracks of $V_{C}$ and $V_{G}^{r}$ for $N_{R}=6000$, $N_{G}=2500$, $D_{G}=0.002\mu m^{2}s^{-1}$, $k_{on}=0.0167s^{-1}nM^{-1}$, $k_{off}=0.1s^{-1}$, $K_{d}=6nM$. (**B**) Temporal tracks of $V_{C}$ and $V_{G}^{r}$ for $N_{R}=375$ using parameters in (A). **(C)** Cumulative distribution functions of angular error comparing gradient sensing efficiency of active G protein in the ratiometric scenario to combined receptor. $N_{R}=[375,3000,6000]$ using parameters in (A). (**D**) Comparison of noise-to-signal ratio with theoretical Cramér-Rao bound which applies to the estimate $V_{C}$. For low values of $N_{R}$, the noise-to-signal ratio for $V_{G}^{r}$ is below this theoretical bound, suggesting that $V_{G}^{r}$ utilizes more information than is present in receptor states at a fixed time. $N_{R}=[188,375,750,1500,3000,6000]$ using parameters in (A)

We next compared the performance of $V_{G}^{r}$ to that of the combined receptor vector $V_{C}$. In simulations with many receptors (large $N_{R}$), the receptor-based vector $V_{C}$ performed better than $V_{G}^{r}$ (Fig.3A). This was expected, as the smaller number of G proteins $\left(N_{G}=2500\right)$ cannot retain all directional information from the larger number of receptors $\left(N_{R}=6000\right)$. However, as $N_{R}$ decreased, $V_{C}$ became increasingly susceptible to stochastic noise. Remarkably, $V_{G}^{r}$ retained its performance at lower $N_{R}$ values (Fig.3A, B), so that below some threshold in receptor count (approx. $N_{R}<1280$), $V_{G}^{r}$ actually outperformed $V_{C}$ (Fig. 3C). Even more unexpectedly, for sufficiently low $N_{R}$ (approx. $N_{R}<800$), $V_{G}^{r}$ outperformed the accuracy limit given by the theoretical Cramér–Rao bound for $V_{C}$ with the same number of receptors (Fig.3C). The Cramér-Rao bound (3) applies only to direction estimates based on instantaneous receptor data (i.e., $V_{C}$, $V_{R}$, $V_{RL}$) and need not apply to $V_{G}^{r}$ or $V_{G}^{c}$; our finding shows that the accuracy of $V_{G}^{r}$ in this regime cannot be matched by any unbiased estimator based only on instantaneous receptors states at a single fixed time. While these simulations were carried out using custom code written in MATLAB 2024a, the surprising results were verified independently using the particle-based simulator SMOLDYN (Figures S1 and S2). Thus, ratiometric G protein signaling can continue to provide accurate directional information despite receptor scarcity.

### C. Ratiometric sensing’s enhanced gradient estimation arises from robust collective G protein memory

In all parameter regimes that we explored with our simulations, the ratiometric model $V_{G}^{r}$ is more accurate in direction estimation than the classical model $V_{G}^{c}$. In some parameter regimes explored in our simulations, the ratiometric model $V_{G}^{r}$ also outperforms both $V_{RL}$ and $V_{C}$, and it even performs better than the theoretical bound for estimation based on instantaneous receptor states (Fig. 3C). Here we explore the reasons for this superior performance of the ratiometric model.

**FIG. 4.**
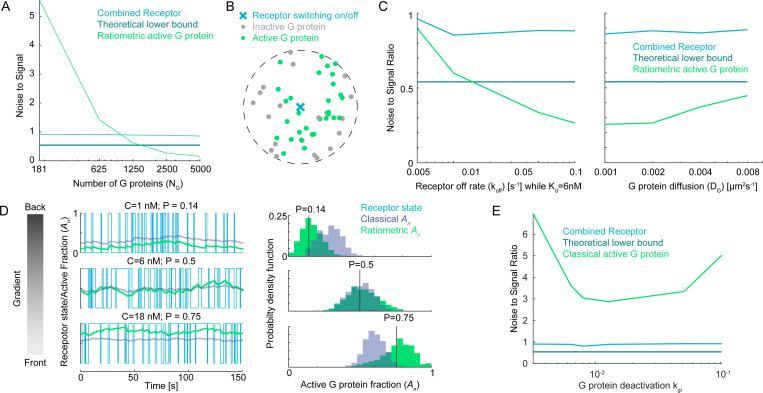
Ratiometric sensing enables G proteins to robustly generate a local average of past receptor states. (**A**) Noise-to-signal ratio for $V_{G}^{r}$, $V_{C}$, and the theoretical lower bound as a function of the number of G proteins. $N_{G}=[181,625,1250,2500,5000]$, $N_{R}=375$, $D_{G}=0.002\mu m^{2}s^{-1}$, $k_{on}=0.0167s^{-1}nM^{-1}$, $k_{off}=0.1s^{-1}$, $K_{d}=6nM$. (**B**) Cartoon of a single receptor surrounded by G proteins that have recently inherited the receptor’s state. For ratiometric signaling, G proteins can inherit state from ON or OFF receptors. For classical signaling, G proteins inherit state from ON receptors, but switch off at basal rate $k_{gi}$.(**C**) Gradient sensing accuracy (NSR) as a function of receptor switching rate (left: $k_{off}=[0.005,0.01,0.05,0.1]\;s^{-1}$) and G protein diffusion constant (right: $D_{G}=[0.001,0.002,0.004,0.008]\;\mu m^{2}s^{-1}$). $N_{G}=2500$, $N_{R}=375$. (**D**) Left: Temporal traces of the active G protein fraction for ratiometric and classical signaling at three positions along a gradient corresponding to different mean ligand concentrations: back $\left(C=1\;nM,p_{R}=0.14\right)$, middle $\left(C=6\;nM,p_{R}=0.5\right)$, and front $\left(C=18\;nM,p_{R}=0.75\right)$. The classical model is tuned to match ratiometric at $p_{R}=0.5$. Right: Histograms of the active G protein fraction showing that the classical model deviates from $p_{R}$ at the front and back, while the ratiometric model remains unbiased across all three regions. (**E**) Sweeping through different values of $k_{gi}$ in the classical model shows optimal performance that does not cross instantaneous theoretical limits. $N_{G}=2500$, $N_{R}=375$. $k_{gi}=[0.0032,0.0064,0.0080,0.0128,0.0512,0.1024]$

The observation that $V_{G}^{r}$ may perform better than both $V_{RL}$ and $V_{C}$, even better than the theoretical bound for estimation based on instantaneous receptor states, suggests that the distribution of active G proteins must carry more information than the instantaneous distribution of receptors alone. Because receptors and G proteins occupy binary states, the G protein population can only carry additional information if $N_{G}>N_{R}$. Indeed, reducing the number of G proteins lowers the accuracy of $V_{G}^{r}$ (Fig. 4A).

We propose that when $N_{G}>N_{R}$, the collection of G proteins captures additional information by repeatedly sampling receptor states over time, thus storing past receptor states. The accuracy of the direction estimate $V_{G}^{r}$ depends on the level of correlation in G protein states, which is governed by two key time scales. The first is the characteristic timescale of receptor state switching, $\lambda^{-1}$, where

(5) $$\lambda =k_{\text{on}}C+k_{\text{off}},$$

$k_{\text{on}}$ and $k_{\text{off}}$ are on/off rates of ligand binding, and C is the local ligand concentration. The second is the timescale of diffusive encounter, $\tau_{\mathrm{dif}}$, which is the mean time between G protein-receptor collisions. This depends on the G protein diffusion constant $\left(D_{G}\right)$ and receptor number $\left(N_{R}\right)$:

(6) $$\tau_{\text{dif}}\propto \frac{L^{2}}{N_{R}D_{G}}\ln\left(\frac{\delta }{r_{*}}\right),$$

where L is the cell’s linear dimension (e.g., radius), $r_{*}$ is the receptor interaction radius, and δ is the typical receptor spacing. In the ratiometric model, it is the product $\lambda \tau_{\text{dif}}$ that determines the degree to which the G protein states are correlated.

We consider a simplified model with n G proteins diffusing in the neighborhood of a single receptor that stochastically switches between active and inactive states, switching to active at rate $k_{\text{on}}C$ and to inactive at rate $k_{\text{off}}$ (Fig.4B). As the receptor is switching states, the n G proteins encounter the receptor at random intervals with mean collision time $\tau_{\text{dif}}$. In the ratiometric version of this simplified model, G proteins activate upon encountering an active receptor but deactivate only upon collision with an inactive receptor. In this case, the mean fraction of active G proteins $\left(A_{n}\right)$ is the same as the fraction of time the receptor is on: $E\left[A_{n}\right]=p_{R}$, but the variance of $A_{n}$ changes with $\tau_{\text{dif}}$ and λ. A lower value of λ would lead to the receptor spending more time in a given state, causing states of G proteins to become more correlated. We derive an expression for variance of $A_{n}$ (see Supplement):

(7) $$\mathrm{Var}\left(A_{n}\right)=p_{R}\left(1-p_{R}\right)\left[\frac{1}{n}+\left(1-\frac{1}{n}\right)\frac{1}{1+\lambda \tau_{\text{dif}}}\right].$$

Thus, faster receptor switching (larger λ) or slower G protein diffusion (larger $\tau_{\text{dif}}$) raises $\lambda \tau_{\text{dif}}$, which lowers $\mathrm{Var}\left(A_{n}\right)$ and improves the accuracy of estimating $p_{R}$. In Section 4 of the Supplement, we explain how this mechanism works when there are multiple receptors distributed across the cell surface, and we show how the timescales $\lambda^{-1}$ and $\tau_{\text{dif}}$ affect the reliability of the direction estimate $V_{G}^{r}$. In our simulations, increasing λ (by increasing $k_{off}$ while keeping $K_{d}$ constant) leads to improved gradient sensing accuracy (lower NSR) (4C left), while increasing $D_{G}$ leads to degraded accuracy (higher NSR) (4C right).

This analysis supports the striking observation that the noise-to-signal ratio of $V_{G}^{r}$ is relatively insensitive to $N_{R}$ across a range of values of $N_{R}$ that extends well below $N_{G}$. Figure 2E, Figure 2C, and Figure 3D each demonstrate this insensitivity over a broad range. In Figure 3D, at low receptor number, $V_{G}^{r}$ maintains a low noise-to-signal ratio—even below what is theoretically achievable for $V_{C}$ at the same $N_{R}$ (i.e., below the Cramér–Rao bound for $V_{C}$). This occurs because historical sampling of multiple receptor states enables $V_{G}^{r}$ to encode more information than is available in instantaneous receptor states. In Figure 4A, as $N_{G}$ increases (with $N_{R}$ fixed), the estimate $V_{G}^{r}$ becomes more accurate than $V_{C}$, the noise-to-signal ratio of $V_{G}^{r}$ ultimately dropping below the Cramér–Rao bound for $V_{C}$.

In principle, the classical model $V_{G}^{c}$ could also benefit from this time averaging, yet we observe that $V_{G}^{c}$ always performs worse than $V_{G}^{r}$. The reason for this difference in performance has to do with a dose-response bias that is inherent in the classical model. For concentration estimation (rather than gradient direction estimation), this bias was observed already in [35]. For the ratiometric model, each G protein’s state corresponds to the state of the receptor it most recently encountered. Consequently, the probability $p_{G}^{r}(x)$ that a G protein at position x is active (in the ratiometric model) corresponds approximately to $p_{R}(x)$, the probability that a receptor at x is active. For the classical model, where a G protein’s state is reset to inactivate at rate $k_{gi}$, the probability $p_{G}^{c}(x)$ that a G protein at x is active depends on how many receptors it encountered since its last resetting event. This number of encounters depends on $k_{gi}$ and on the diffusive time scale $\tau_{\text{dif}}$. In the supplement we derive the formula

(8) $$p_{G}^{c}(x)\approx \frac{p_{R}(x)E[N]}{1+p_{R}(x)E[N]}$$

where $E[N]=\tau_{\text{off}}/\tau_{\text{dif}}$ is the mean number of encounters, and $\tau_{\text{off}}=k_{gi}^{-1}$. A consequence of this is that $p_{G}^{c}$ is not equal to $p_{R}$ across the cell, and typically the gradient of $p_{G}^{c}$ is significantly flatter than that of $p_{R}$. Since the NSR for direction estimation is inversely proportional to the square of the ligand gradient (e.g., $1/b^{2}$ in (4)), this flattening of the gradient of $p_{G}^{c}$ relative to that of $p_{R}$ implies a loss of accuracy in direction estimation for $V_{G}^{c}$.

To see how this bias effect may affect gradient sensing, we compared simulations of the ratiometric and classical versions of the simplified model described above. In the classical version, the n G proteins become activated upon collision with an active receptor and deactivate spontaneously at a baseline rate $k_{gi}$. To compare these models, we examined the active G protein fraction $\left(A_{n}\right)$ at three positions across the cell corresponding to different ligand concentrations: back (low concentration, $p_{R}=0.14$), middle $\left(p_{R}=0.5\right)$, and front (high concentration, $p_{R}=0.75$) (Fig. 4D). At the middle position, where $p_{R}=0.5$, we can tune $k_{gi}$ so that the classical model matches the ratiometric model, with both accurately reflecting the receptor state. However, at the front and back, where receptors experience higher or lower ligand concentrations, the classical model with this fixed $k_{gi}$ deviates from $p_{R}$, while the ratiometric model maintains an unbiased estimate across all three regions without any parameter adjustment. Consequently, the ratiometric model produces a steeper gradient from front to back than the classical case.

This observation suggests the possibility of optimally tuning $k_{gi}$ to improve gradient sensing in the classical model. We therefore swept through different values of $k_{gi}$ in our full cellular gradient-sensing simulations (Fig. 4E). While we identified an optimal $k_{gi}$ value that minimized the noise-to-signal ratio, this optimized classical model still performed worse than the combined receptor estimate $V_{C}$ and failed to surpass the instantaneous theoretical bound, which is markedly inferior to the ratiometric model (Fig. 3D). Thus, ratiometric signaling enables robust collective memory through unbiased local averaging of receptor states, allowing cells to surpass the instantaneous theoretical limit for gradient detection.

### D. Responsiveness to changing gradients

In the preceding discussion, we assumed that ligand gradients were stable over time. However, this is not always the case: neutrophils chase motile bacteria, social amoebae collectively generate fruiting bodies by following dynamically shifting cAMP signals, and budding yeast track their mating partners as both cells grow [10, 40, 41]. While the robust collective memory encoded in G protein states by ratiometric signaling improves accuracy at steady state, it may slow responses to changing gradient directions.

**FIG. 5.**
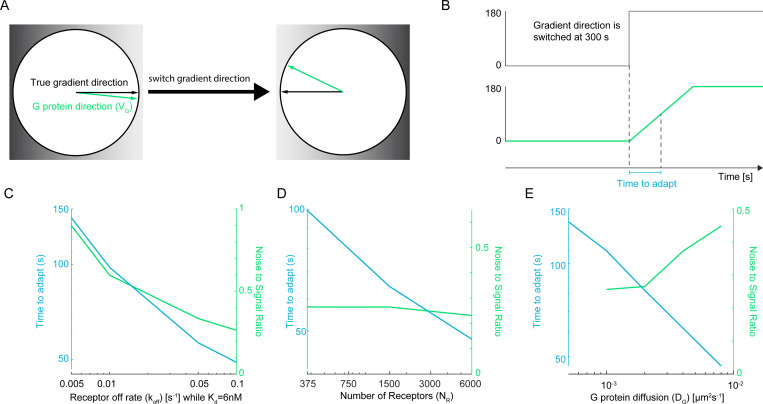
Adaptation to changing gradients. Default simulation parameters: $N_{R}=375$, $N_{G}=2500$, $k_{\text{off}}=0.1\;{\text{s}}^{-1}$, $K_{d}=6\;\text{nM}$, and $D_{G}=0.002\;\mu {\text{m}}^{2}{\text{s}}^{-1}$. (**A**) Cartoon demonstrating simulation where the true gradient direction (black arrow) is flipped by 180°. The G protein direction vector $V_{G}$ (green arrow) eventually re-orients to match the new gradient. (**B**) Cartoon demonstrating how adaptation time of gradient estimate by G proteins is calculated. Top: The environmental gradient direction is switched at t=300 s from 0° to 180°. Bottom: The direction of the averaged trace derived from 1000 G protein vectors $\left(V_{G}^{r}\right)$ lags before reaching the new steady state. The time to adapt is defined as the interval required to cross 90° toward the new directional equilibrium. (**C–E**) Parameter sensitivity of adaptation speed and sensing accuracy. Blue curves (left y-axis) indicate time to adapt; green curves (right y-axis) indicate the steady-state noise-to-signal ratio (NSR). (**C**) Sweeping receptor off-rate $\left(k_{\text{off}}\right)$ from 0.005 to 0.1 s^−1^ shows that faster receptor kinetics improve both adaptation speed and directional accuracy. (**D**) Sweeping the number of receptors $\left(N_{R}\right)$ from 375 to 6000 shows that higher receptor density significantly reduces the time to adapt while maintaining a relatively robust NSR. (**E**) Sweeping G protein diffusion $\left(D_{G}\right)$ from 10^−3^ to 10^−2^ *μ*m^2^s^−1^ shows that faster diffusion accelerates adaptation but leads to a higher NSR, highlighting a fundamental trade-off between response speed and spatial precision.

To assess how different parameters affect a cell’s ability to adapt to changing gradients, we performed simulations in which we allowed $V_{G}^{r}$ to reach steady state in a gradient and then instantaneously reversed the gradient direction by 180 degrees (Fig. 5A). We quantified responsiveness by measuring the time required for $V_{G}^{r}$ to cross the 90-degree threshold toward the new gradient direction (Fig. 5B).

We first examined the effect of receptor switching kinetics by varying both ligand off-rate $k_{\text{off}}$ and on-rate $k_{on}$ while maintaining a constant $K_{d}$ (Fig. 5C). While $k_{\text{on}}$ is physically capped by the rate of diffusional encounters, our parameters remain biologically plausible; at $K_{d}=6$ nM, the maximum $k_{\text{off}}=0.1\;{\text{s}}^{-1}$ corresponds to $k_{\text{on}}\approx 1.67\times 10^{7}\;{\text{M}}^{-1}\;{\text{s}}^{-1}$, which is well within the Smoluchowski limit. Increasing $k_{\text{off}}$ from 0.005 to 0.1 s^−1^ reduced both the time to adapt and the steady-state noise-to-signal ratio. These dual benefits can be understood through the receptor switching rate λ (Equation 5): increasing $k_{\text{off}}$ increases λ, which directly accelerates receptor state transitions and reduces the time to achieve statistical equilibrium after the gradient direction is reversed. Simultaneously, higher λ increases the product $\lambda \tau_{\text{dif}}$; as we have discussed, this reduces correlation in downstream G protein states, and thus improves steady-state directional accuracy.

Increasing receptor density also accelerated adaptation to new gradient directions (Fig. 5D). Higher $N_{R}$ reduces the mean time between G protein–receptor encounters $\left(\tau_{\text{dif}}\right)$, allowing G proteins to update their states more frequently and thereby adapt more quickly to the reversed gradient. As we have already noted, the noise-to-signal ratio of $V_{G}^{r}$ (at steady state) may be relatively insensitive to changes in $N_{R}$ over a certain range, depending on $N_{G}$ (e.g., as $N_{R}$ increased from 375 to 6000 in Fig. 5D).

In contrast to receptor kinetics and density, increasing the G protein diffusion constant $D_{G}$ presented a trade-off between adaptation speed and steady-state accuracy (Fig. 5E). Faster diffusion reduces $\tau_{\text{dif}}$, enabling more frequent G protein–receptor encounters and thereby accelerating adaptation to the new gradient direction. However, unlike increasing $N_{R}$, faster diffusion of G protein increases correlation in G protein states and increases the noise-to-signal ratio of $V_{G}^{r}$ at steady state.

Together, these results reveal distinct strategies by which cells could tune their responsiveness to changing gradients. Faster receptor kinetics provide the most favorable outcome, simultaneously enhancing both steady-state accuracy and adaptation speed. Increased receptor density primarily accelerates adaptation with minimal impact on steady-state performance. Higher G protein diffusion offers faster adaptation but compromises spatial precision—a trade-off that cells may exploit depending on whether their environment demands accurate gradient tracking or rapid reorientation.

## III. DISCUSSION

### A. Ratiometric signaling in the budding yeast system

Gradient decoding is most challenging when cells are small, gradients are shallow, and chemical concentrations are low, but yeast cells find mates despite encountering all of these challenges. An effective strategy to extract signals from noisy measurements would be to integrate concentration measurements over time, allowing time averaging to reduce the noise (fluctuations) without affecting the signal (gradient). However, once a chemical has bound to a cell-surface receptor, diffusion of the receptor-ligand complex would degrade the information about the location where the binding took place. Such blurring can limit the interval over which time-averaging is effective for directional sensing[35]. This is particularly problematic for systems like yeast pheromone sensing, where ligand-receptor complexes can persist for several minutes. Interestingly, yeast cells severely limit receptor diffusion (D<0.0005 *μm*^2^/*s*), perhaps mitigating this problem [33]. However, with very slow receptor diffusion, receptors often accumulate non-uniformly on one side of the cell [33, 42–44]. Non-uniform receptor distributions in turn can lead to spatial patterns of ligand-receptor complexes that do not accurately reflect the spatial distribution of ligands in the environment.

Yeast cells appear to use a ratiometric sensing strategy to compensate for non-uniform receptor distributions [33]. Ligand binding to receptors promotes activation of G proteins that are then turned off by RGS proteins (Sst2 in yeast). In classical signaling models, G protein inactivation occurs in a receptor-independent manner, but in yeast cells the RGS protein is bound to inactive receptors [42]. Thus, whereas active receptors promote signaling, inactive receptors inhibit signaling, and the net signal reflects the ratio of active to inactive receptors [34]. If receptors are concentrated in some region of the cell surface, both active and inactive receptors may be locally enriched, so ratiometric sensing can allow the cell to accurately infer the local ligand distribution by compensating for the local receptor abundance. Interestingly, we find that, in addition to extracting information from both active and inactive receptors, ratiometric sensing could also better enable a cell to interpret temporally averaged receptor states over time.

### B. Noise in concentration measurement from ratiometric signaling

In principle, ratiometric signaling could be a widely applicable strategy to improve the detection of spatial gradients, especially when gradient concentrations are near the ligand-receptor $K_{d}$. It seems intuitively plausible that a cell capable of using information about the spatial distributions of inactive as well as active receptors would have a firmer basis to infer the spatial distribution of ligand than a cell whose only information comes from the active receptors. However, as discussed in the Supplement and shown in [38, 39], ratiometric signaling can be less accurate at sensing absolute concentration. Nevertheless, we find that despite being worse at sensing ligand concentration, the ratiometric strategy provides more accurate gradient sensing by enabling acquisition of information from both active and inactive receptors as well as locally averaging receptor signals over time.

We show that ratiometric signaling outperforms classical signaling, even at very low receptor numbers when less than 0.02% of the cell surface is covered in receptors. Notably, these conditions resemble scenarios where cells initially populate their membranes with specific receptors—such as in lymphocytes like B cells, which upregulate receptors upon transitioning between germinal center zones; and in various developmental contexts where cells switch states and begin expressing new receptors [45, 46]. In such scenarios, ratiometric signaling could enable reliable gradient detection with lower receptor density, all while resisting misdirection by local fluctuations in receptor localization or ligand availability.

### C. Mechanism underlying gradient detection accuracy by ratiometric signaling

One of our most intriguing observations was that ratiometric signaling could outperform the theoretical bound for gradient direction estimates that use only receptor snapshots at a single moment in time. This surprising advantage arises because downstream molecules—specifically, diffusing G proteins—act as a form of collective molecular memory. As each G protein updates its activity state only upon encountering a receptor, it encodes receptor information from an earlier time point. A population of G proteins that do this for both active and inactive receptors can smooth out short-lived fluctuations and enhance the fidelity of direction estimates. In effect, G proteins collectively time-average receptor states, allowing the cell to extract more robust spatial information than would be possible from instantaneous receptor states alone.

In ratiometric signaling, two parameters critically balance directional accuracy and adaptive speed: the switching rate (λ) of receptor-ligand binding and the diffusion coefficient $\left(D_{G}\right)$ of G proteins. Increasing λ enhances collective local averaging by G proteins (Fig. 4E (left) and 5C), improving steady-state gradient detection, and promotes faster adaptation to shifting gradients (Fig. 5C). In contrast, raising $D_{G}$ weakens local averaging and diminishes steady state-gradient detection (Fig. 4E (right) and 5E). But higher $D_{G}$ accelerates adaptation to changing signals, as more frequent receptor–G protein collisions swiftly update G protein states in response to new external conditions (Fig. 5C). Together, these trade-offs highlight how ratiometric signaling can be tuned to optimize either accuracy or speed, as required by the biological context.

### D. Broader relevance of ratiometric signaling in responding to directional cues

It is not known whether receptors other than GPCRs can engage ratiometric signaling pathways. However, the principle underlying the averaging of upstream signals by more abundant downstream components may extend beyond receptor signaling, particularly in systems where both active and inactive states of upstream proteins convey meaningful information. In MAPK cascades, downstream kinases often exceed the abundance of their upstream regulators, exemplified by Raf in mammalian ERK pathways [47], B-Raf in cancer signaling contexts [48], and Ste11 in yeast mating responses [49]. These systems possess features that suggest the potential for ratiometric measurement-driven averaging to enhance accuracy and robustness.

Overall, our work suggests that ratiometric signaling mechanisms provide a powerful framework for cells to interpret directional receptor information and mitigate noise. The ability to sample both active and inactive states, combined with downstream emergent molecular memory, enables more accurate direction finding than that achieved by classical signaling mechanisms. These results offer testable predictions for experimental systems and may help clarify why some cells have a higher abundance of downstream proteins in signaling cascades.

## Supplementary Material

Supplement 1

## Figures and Tables

**FIG. 6.**
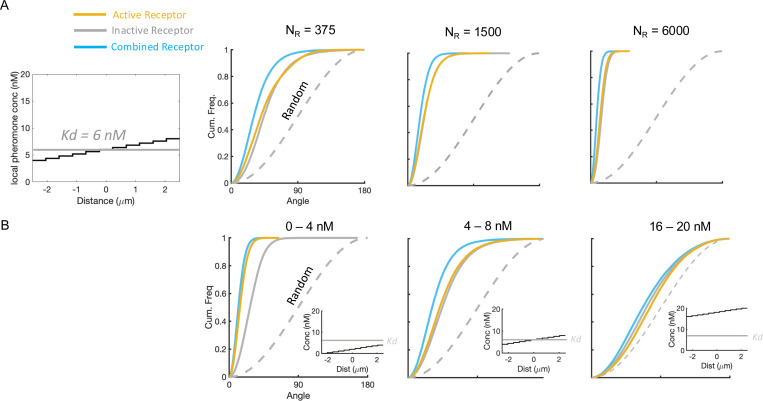
Replicating results in comparison of receptor signals using Smoldyn. **A)** Angular distribution of receptors with the same pheromone gradient centered around $K_{d}$ but under different receptor number. Corresponds to Fig. 1B. n=10 cells. **B)** Angular distribution of receptors with the same receptor number but under different heights of pheromone gradients. Corresponds to Fig. 1D. n=50 cells.

**FIG. 7.**
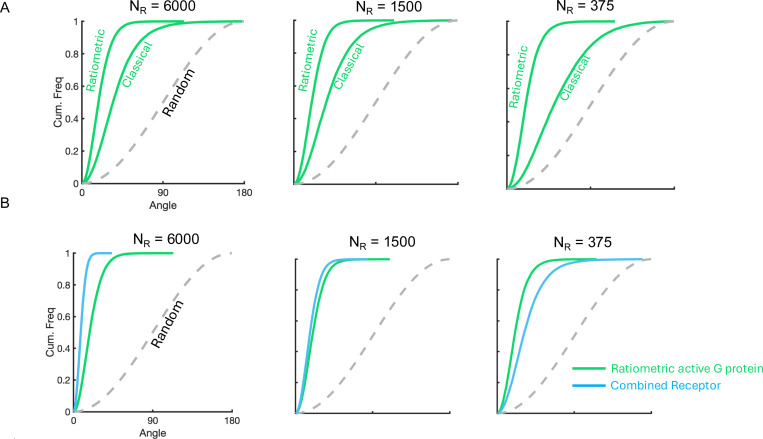
Replicating results in comparison between ratiometric and classical active G protein signal and combined receptor signal using Smoldyn. **A)** Angular distributions comparing gradient sensing between ratiometric and classical active G proteins. Corresponds to Fig. 2D. n=10 cells. **B)** Angular distributions comparing gradient sensing between ratiometric active G proteins and combined receptors. Corresponds to Fig. 3C. n=10 cells.

**FIG. 8.**
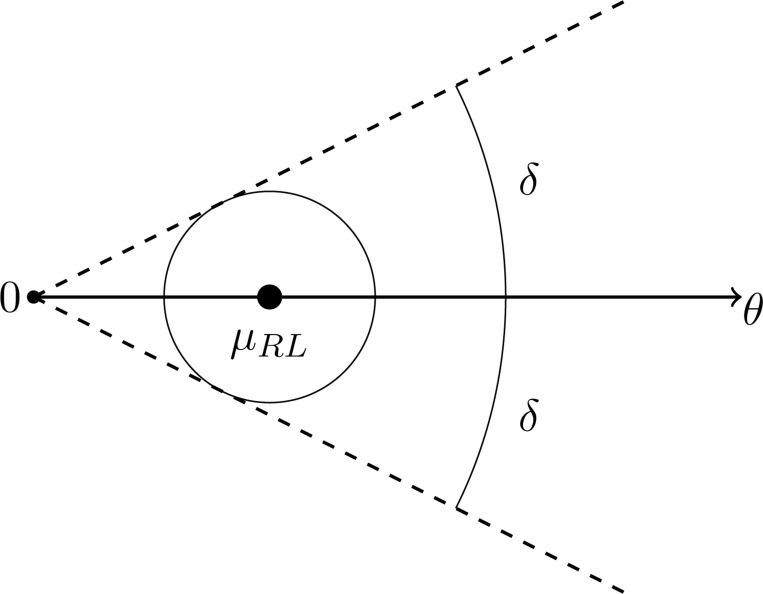
The figure illustrates the cone $C_{\delta ,\theta }$. The ball centered at μ has radius r=|μ|sin(δ). The condition (B17) guarantees that the standard deviation of V is less than $r/\sqrt{2}$, so that V lies in the ball with probability at least 1/2.

**FIG. 9.**
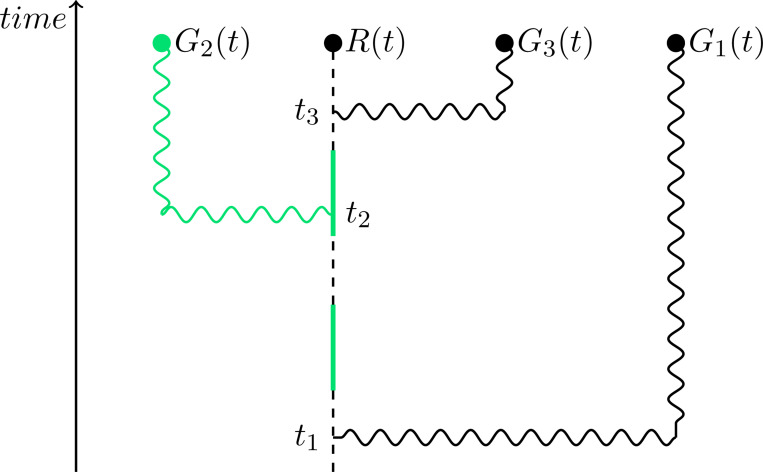
This figure illustrates how G proteins record the historical state of a receptor. The states of G proteins $G_{1}$, $G_{2}$, and $G_{3}$ at time t were obtained through interaction with the same receptor, but at different earlier times. The protein $G_{1}$ records the receptor state from time $t_{1}<t$; we say at that time t the age of $G_{1}$ is $t-t_{1}$. Protein $G_{2}$ records the receptor state from time $t_{2}<t$; at time t the age of $G_{2}$ is $t-t_{2}$. Even though each protein last interacted with the same receptor, the states they record at time t may differ: $G_{1}$ and $G_{3}$ are inactive, while $G_{2}$ is active.

**FIG. 10.**
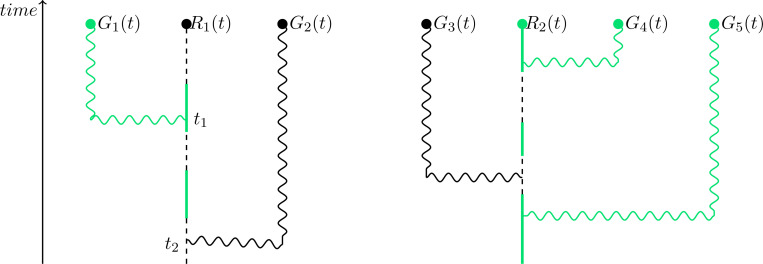
This figure illustrates the most recent receptor-interactions among a collection of five G proteins. The states of G proteins $G_{1}$ and $G_{2}$ at time t were obtained through interaction with the same receptor $R_{1}$. Proteins $G_{1}$ and $G_{2}$ are linked to receptor $R_{1}$. However, proteins $G_{3}$, $G_{4}$, $G_{5}$ are linked to receptor $R_{2}$, since their most recent receptor interaction was with receptor $R_{2}$.

**FIG. 11.**
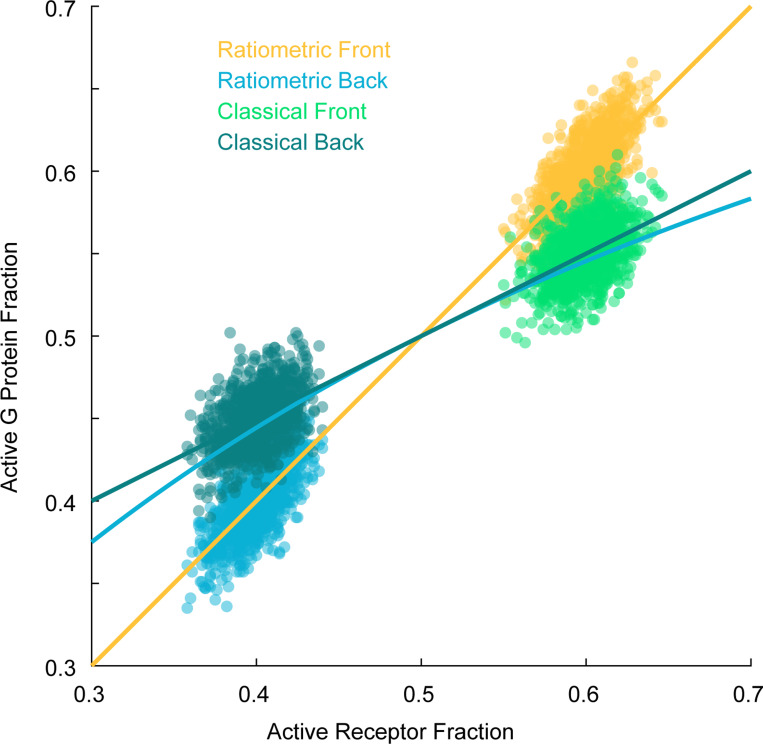
Empirical joint distribution of $\left(R_{f}^{+}/N_{R},G_{f}^{+}/N_{G}\right)$ and $\left(R_{b}^{+}/N_{R},G_{b}^{+}/N_{G}\right)$, the active receptor and active G protein fractions at front and back of cell. Front of cell: ratiometric (yellow) and classical (green). Back of cell: ratiometric (blue) and classical (dark green). $N_{R}=N_{G}=1000$. ϵ=0.1. The red line is the (linear) activation function ℓ(r)=r, which applies to the ratiometric case. The blue and black lines are the activation functions r/(r+1/2) and its linearization $\frac{1}{2}+\frac{1}{2}(r-\frac{1}{2})$, which applies to the classical model (see equation (E2)).

**FIG. 12.**
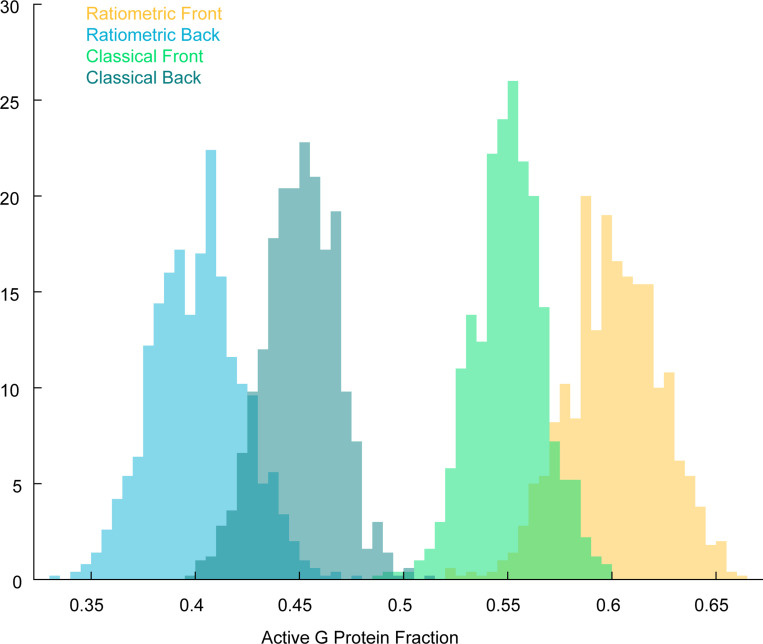
Distributions (histograms) of $G_{f}^{+}/N_{G}$ and $G_{b}^{+}/N_{G}$, the active G protein fraction at the front and back of cell. Front of cell: ratiometric (yellow) and classical (green). Back of cell: ratiometric (blue) and classical (dark green). These correspond to marginal distributions from the joint distribution plot above, Figure 11. $N_{R}=N_{G}=1000$. ϵ=0.1

**FIG. 13.**
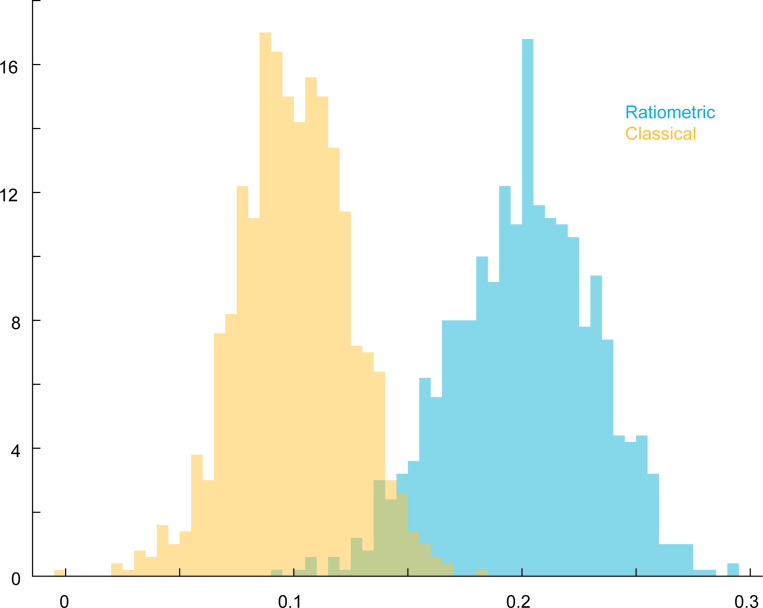
Distributions (histograms) of $\left(G_{f}^{+}-G_{b}^{+}\right)/N_{G}$, the active G protein gradient from front to back of cell, for Ratiometric (blue) and Classical (yellow).

**FIG. 14.**
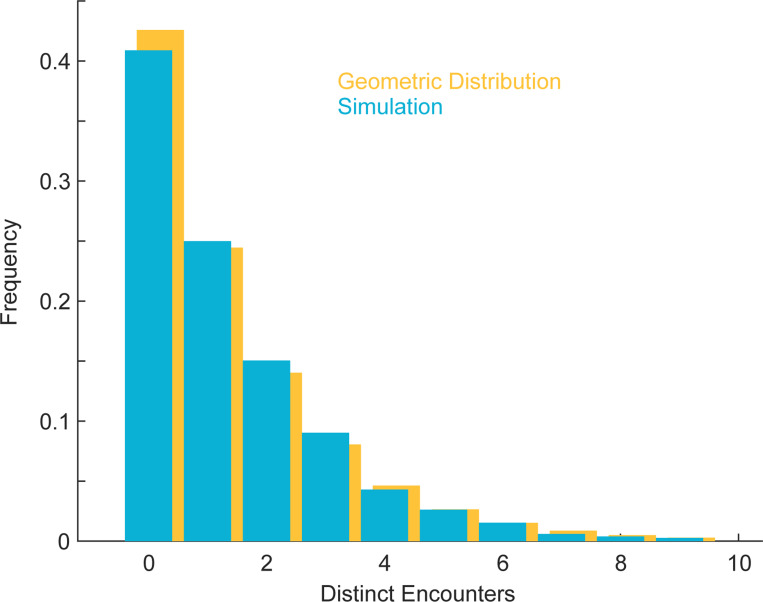
**Blue:** Empirical distribution of N, the number of distinct receptor encounters by a G protein since its most recent state-reset time. $N_{R}=375$, $D_{G}=0.002{\left(\mu m\right)}^{2}/sec$, $k_{go}=0.0083{\left(sec\right)}^{-1}$. The mean number of encounters is E[N]=1.28. Fraction of proteins not encountering a receptor: 0.42. Mean time to first encounter: 79.9. 20,000 proteins were simulated, for a single realization of the receptor configuration. **Yellow:** For comparison, the Geometric distribution given by (F1).

**FIG. 15.**
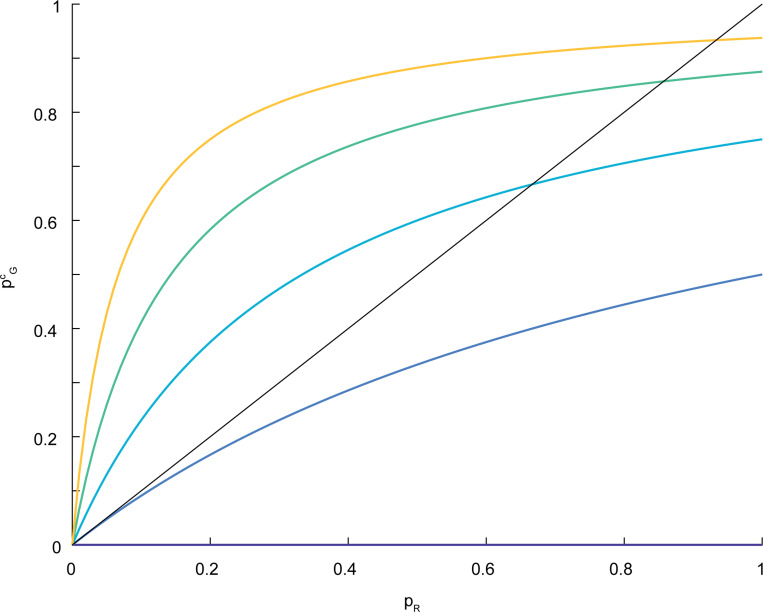
Graph of $p_{G}^{c}$ versus $p_{r}$, for different values of $E[N]=2^{k}-1$, k=0,…,4. For comparison, the straight line has slope 1.

## Data Availability

The data and simulation code that support the findings of this study are openly available in the following GitHub repository: https://github.com/DebrajGhose/ratiometric-sensing-robust.
