# Supplementary material for: Ratiometric signaling produces robust temporal integration for accurate cellular gradient sensing
Source: bioRxiv. 2026 Feb 18:2025.04.18.649595. Preprint. [Version 2] doi: 10.1101/2025.04.18.649595 (PMC12934627; doi:10.1101/2025.04.18.649595)
Supplement: Supplement 1 [file NIHPP2025.04.18.649595v2-supplement-1.pdf]

bioRxiv preprint doi: https://doi.org/10.1101/2025.04.18.649595; this version posted February 18, 2026. The copyright holder for this preprint (which was not certified by peer review) is the author/funder, who has granted bioRxiv a license to display the preprint in perpetuity. It is made available under aCC-BY 4.0 International license.

Appendix A: Simulation Framework

The simulation models the interactions between receptors and G proteins on a cell surface, taken to be a sphere $\mathcal{S}$ in three dimensions. The simulation focuses on their activation and inactivation dynamics under a pheromone gradient.

On the surface of the sphere, receptors are positioned randomly; their locations are fixed and do not change over time. Receptors also switch state randomly and independently of each other; they can be either ligand-bound (active) or unbound (inactive). The rate of activation depends on the local ligand concentration, which is taken to be time-independent.

The G proteins are also positioned randomly over the surface of the sphere, independently and uniformly distributed. Unlike the receptors, however, each G protein performs a diffusive random walk along the surface of $\mathcal{S}$. G proteins in the simulation can exist in either an active or inactive state. The state of a G protein is influenced by its interaction with nearby receptors and its inherent deactivation rate. G proteins switch state according to either the classical model or the ratiometric model. In both models, a G protein becomes activated upon encountering an active receptor. The difference in the two models lies in the mechanism for deactivation. In the classical model, a G protein becomes deactivated randomly at the inherent deactivation rate. In the ratiometric model, a G protein becomes deactivated by encountering an inactive receptor.

We use the following nomenclature:

bioRxiv preprint doi: https://doi.org/10.1101/2025.04.18.649595; this version posted February 18, 2026. The copyright holder for this preprint (which was not certified by peer review) is the author/funder, who has granted bioRxiv a license to display the preprint in perpetuity. It is made available under aCC-BY 4.0 International license.

$\mathcal{S}$	cell surface, a sphere in $\mathbb{R}^3$, centered at the origin
L	radius of the cell (2.5 μm for a yeast cell)
x	cartesian coordinate for a position on the sphere $\mathcal{S}$
θ	true direction of pheromone concentration gradient, $\theta \in \mathbb{R}^3$, $\lVert\theta\rVert = 1$
$C(x)$	local ligand concentration at position x on cell surface
b	gradient steepness parameter for ligand concentration, $b \in (0, 1)$
K_d	dissociation constant for pheromone-receptor binding (6 nM for yeast)
k_{off}	rate of receptor deactivation or ligand unbinding (typically $0.1s^{-1}$ in our simulations)
k_{on}	rate constant for receptor activation, $k_{\text{on}} = k_{\text{off}}/K_d$
N_R	total number of receptors (from 375 to 6000 in our simulations)
$X_1, \ldots, X_{N_R}$	locations of the receptors on $\mathcal{S}$
$R_1, \ldots, R_{N_R}$	states of receptors, either 0 (inactive, unbound) or 1 (active, bound)
N_G	total number of G proteins on $\mathcal{S}$ (from 181 to 5000 in our simulations)
$Y_1, \ldots, Y_{N_G}$	locations of the G proteins on $\mathcal{S}$
$G_1, \ldots, G_{N_G}$	states of the G proteins, $G_j \in \{0, 1\}$.
r_*	activation radius for activation of G protein by an active receptor (5 nm)
D_G	diffusion constant for G protein (from 0.001 to 0.008 $\mu m^2/s$ in our simulations)
$k_i = 0$	G protein inactivation rate constant for ratiometric model
k_i'	G protein inactivation rate constant for nonratiometric model

1. Receptor dynamics

The receptor configuration at time t is characterized by the pairs $\{(X_k, R_k(t))\}_{k=1}^{N_R}$. The positions $\{X_k\}_{k=1}^{N_R}$ are sampled independently and uniformly on $\mathcal{S}$. The receptor positions are assumed to not change with time (we neglect receptor diffusion). Given a receptor at position $X_k = x$, the receptor state $R_k(t) \in \{0, 1\}$ changes as a Markov process with rates:

$$0 \mapsto 1 \qquad \text{at rate } k_{\text{on}} C(x), \tag{A1}$$

$$1 \mapsto 0 \qquad \text{at rate } k_{\text{off}}. \tag{A2}$$

The receptor states are initialized according to the stationary distribution. Therefore, at any time, the probability that a receptor at location $X_k = x$ is active is:

$$p_R(x) = \mathbb{P}(R_k(t) = 1 \mid \; X_k = x) = \frac{C(x)}{C(x) + K_d}, \qquad K_d = k_{\text{off}}/k_{\text{on}}.$$

bioRxiv preprint doi: https://doi.org/10.1101/2025.04.18.649595; this version posted February 18, 2026. The copyright holder for this preprint (which was not certified by peer review) is the author/funder, who has granted bioRxiv a license to display the preprint in perpetuity. It is made available under aCC-BY 4.0 International license.

The ligand concentration depends on the direction vector θ, the true gradient direction. We will write $C = C(x,\theta)$ to emphasize this dependence on θ. We take C to have the linear form

$$C(x,\theta) = C_0(1 + b(x\cdot\theta)/L), \quad x \in \mathcal{S}, \tag{A3}$$

where $C_0 > 0$ and $b \in (0,1)$, so that $(1-b)C_0 \le C(x,\theta) \le (1+b)C_0$. Consequently, the probability $p_R(x)$ also depends on θ, and we emphasize this by writing $p_R(x,\theta)$.

As we will explain later, the rate parameter

$$\lambda_0 = k_{\text{on}}C_0 + k_{\text{off}}$$

plays an important role in the model; λ_0^{-1} has the units of $(time)$ and we refer to λ_0^{-1} as the receptor turnover time.

We consider values of N_R in the range 375 to 6000 in our simulations. For a cell radius $L = 2.5\mu m$ and receptor activation radius $r_* = 5nm$, the fraction of area covered by receptor is in the range of 0.0375% to 0.6%.

2. G protein dynamics

The G proteins are initialized independently, uniformly at random on the sphere, at positions $Y_1(0),\ldots,Y_{N_G}(0)$. These positions diffuse over $\mathcal{S}$ in time, independently and with diffusion constant D_G; statistically, their joint spatial distribution is invariant in time. An inactive G protein (state $G_j(t) = 0$) at location $Y_j(t)$ is activated upon interaction with an active receptor. Such activation occurs when $Y_j(t)$ diffuses within radius r_* of an active receptor. The simulation uses a spatial partitioning algorithm (KD-tree) to identify, at each time step, G proteins within a certain reaction radius of any receptor.

Once active ($G_j = 1$), the mechanism for de-activation of G protein is either the ratiometric model or the classical model. In the classical model, an active G protein deactivates at a fixed rate k_i', the inherent deactivation rate, but an active G protein is unaffected by encountering inactive receptors. In the ratiometric model, however, an active G protein deactivates upon diffusing within radius r_* of an inactive receptor and there is no basal deactivation ($k_i = 0$ in the ratiometric model). To compare the ratiometric and classical models, we choose k_i' so that at steady state, the mean fraction of active G protein is the

bioRxiv preprint doi: https://doi.org/10.1101/2025.04.18.649595; this version posted February 18, 2026. The copyright holder for this preprint (which was not certified by peer review) is the author/funder, who has granted bioRxiv a license to display the preprint in perpetuity. It is made available under aCC-BY 4.0 International license.

same as that for the ratiometric model. In particular, we adjust the parameter k_i' for each ligand concentration profile to maintain this comparison.

Depending on the random receptor configuration, it is possible for a G protein to be within the reaction radius of two or more receptors simultaneously. For the ratiometric model, when a G protein is near multiple receptors simultaneously the state (at each time step) of the G protein is matched that of a neighboring receptor chosen uniformly at random (from among those receptors within the reaction radius). Hence, if the majority of nearby receptors are active, the probability increases for the G protein to become active. Conversely, if most nearby receptors are inactive, the likelihood increases for the G protein to become inactive. This dynamic represents a ratiometric response of the G protein to the local receptor state.

3. Simulation methodology and verification

Simulations were performed using custom solvers in MATLAB (v2021b to v2024a) on a Linux-based computing system (Longleaf cluster at UNC Chapel Hill). Simulations were initialized by randomly and uniformly distributing receptor and G protein populations on the surface of a spherical cell. Receptor positions were kept stationary while G proteins performed random walks (Brownian motion) across the cell's surface with a fixed time step and diffusion constant. Receptor activation followed a first-order reaction, with an activation rate determined by the local pheromone concentration prescribed along the cell's x-axis to establish a gradient (Equation (A2) and A 1). Activation and deactivation events were implemented via Monte Carlo sampling. In the classical receptor model, G proteins became active upon encountering active receptors within a specified reaction distance and deactivated at a constant rate. In the ratiometric model, G proteins were activated upon encountering active receptors within the same reaction distance and deactivated upon encountering inactive receptors. In the presence of multiple receptors, the ratio of active to inactive receptors governed the probability of G-protein activation or deactivation. For each set of parameters, 1000 simulations were run, tracking molecular states, positions, and time-resolved changes in receptor and G-protein activation. All MATLAB code for these simulations is available at: https://github.com/DebrajGhose/ratiometric-sensing-robust

To validate our solver, MATLAB simulations from Figures 1, 2D and 3C were replicated in Smoldyn (v2.71) with continuous space and discretized time intervals on a Linux-based

bioRxiv preprint doi: https://doi.org/10.1101/2025.04.18.649595; this version posted February 18, 2026. The copyright holder for this preprint (which was not certified by peer review) is the author/funder, who has granted bioRxiv a license to display the preprint in perpetuity. It is made available under aCC-BY 4.0 International license.

computing system (Longleaf cluster at UNC Chapel Hill). Periodic boundary conditions were applied in both spatial directions. Molecules were modeled as point particles without volumes. Brownian motion of the molecules was simulated using the Euler-Maruyama method. We used a time step of 0.005 secs and recorded the coordinates of receptors and G proteins every 10 secs over 20000 secs. The simulation methods used were consistent with the customized code, except for the setup of the pheromone gradient. As Smoldyn does not support continuously spatially dependent concentration, the cell sphere was divided into 11 vertical compartments along the gradient direction, with each compartment assigned a local pheromone concentration defined as:

$$P_{local,i} = P_{low} + (i-1)\frac{P_{high} - P_{low}}{N-1}$$

where P_{low} and P_{high} represent the lowest and highest pheromone concentrations, respectively. i is the compartment index. $N = 11$ is the total number of compartments. Accordingly, we modeled the receptor activation as a first-order reaction with a local activation rate in the ith compartment defined as:

$$k_{a,i} = \frac{k_d}{K_d} \times P_{local,i}$$

where $k_d = 0.1s^{-1}$ is the dissociation rate between the receptor and the ligand.

All code for Smoldyn simulations are available at: https://github.com/DebrajGhose/ratiometric-sensing-robust Figure 6 and 7 show that Smoldyn simulations produce similar results to those of our custom code.

Appendix B: Estimation model

At a given time t, we use the receptor data and the G protein data to compute various estimates of the direction parameter θ, treated as an unknown quantity. The receptor data at a fixed time are random variables $\{(X_k, R_k(t))\}_{k=1}^{N_R}$. These variables are pairwise independent, and their distribution depends on the parameter θ. Thus, the receptor data may be regarded as N_R independent points in $\mathcal{S} \times \{0,1\}$, and their joint density on $(\mathcal{S} \times \{0,1\})^{N_R}$ is

$$\rho(\{(X_k, R_k)\}, \theta) = \prod_{k=1}^{N_R} \frac{1}{4\pi L^2} \left(p_R(X_k, \theta)\mathbb{I}_{R_k=1} + (1 - p_R(X_k, \theta))\mathbb{I}_{R_k=0}\right). \quad \text{(B1)}$$

bioRxiv preprint doi: https://doi.org/10.1101/2025.04.18.649595; this version posted February 18, 2026. The copyright holder for this preprint (which was not certified by peer review) is the author/funder, who has granted bioRxiv a license to display the preprint in perpetuity. It is made available under aCC-BY 4.0 International license.

The factor $4\pi L^2$ is the surface area of $\mathcal{S}$. The terms $\mathbb{I}_{R=1}$ and $\mathbb{I}_{R=0}$ are indicators of the events $R = 1$ and $R = 0$, respectively.

Using active receptor data, we define

$$V_{RL} = \sum_{k=1}^{N_R} X_k \mathbb{I}_{R_k=1} \tag{B2}$$

where $\mathbb{I}_{R_k=1}$ is the indicator of the event $R_k = 1$. Thus, V_{RL} is the sum of the positions of active receptors only. Similarly, using inactive receptors, we define

$$V_R = \sum_{k=1}^{N_R} X_k \mathbb{I}_{R_k=0}, \tag{B3}$$

and we regard the reflected vector $(-V_R)$ as an estimate of θ, since the gradient of inactive receptor will typically be in a direction anti-aligned with θ. The combined receptor estimate is

$$V_C = V_{RL} - V_R = \sum_{k=1}^{N_R} X_k \left(\mathbb{I}_{R_k=1} - \mathbb{I}_{R_k=0}\right).$$

These V_{RL}, $-V_R$, and V_C are vectors in $\mathbb{R}^3$. From these vectors, direction information is computed by normalizing

$$\bar{V}_C = \frac{V_C}{\|V_C\|}, \qquad \bar{V}_{RL} = \frac{V_{RL}}{\|V_{RL}\|}, \qquad -\bar{V}_R = \frac{-V_R}{\|V_R\|}.$$

Each of these direction estimates is a random unit vector, depending on time (because the receptor states change in time). To compute the distribution of these estimates, we obtain independent samples by re-initializing independently all of the receptor positions and states.

Using the G protein data, we compute the vector

$$V_G = \sum_{j=1}^{N_G} Y_j \mathbb{I}_{G_j=1} \tag{B4}$$

which is the sum of positions of all active G proteins. From this a direction estimate is computed by normalizing:

$$\bar{V}_G = \frac{V_G}{\|V_G\|}.$$

Where appropriate, we will use superscripts V_G^r and V_G^c to further distinguish the cases where the vector (B4) is computed under the ratiometric model versus the classical model, respectively. Like the estimates $\bar{V}_C$, $\bar{V}_{RL}$ and $-\bar{V}_R$, the estimate $\bar{V}_G$ is a random unit vector that depends on time. However, $\bar{V}_G(t)$ is not a function of the receptor data $\{X_k, R_k(t)\}$

bioRxiv preprint doi: https://doi.org/10.1101/2025.04.18.649595; this version posted February 18, 2026. The copyright holder for this preprint (which was not certified by peer review) is the author/funder, who has granted bioRxiv a license to display the preprint in perpetuity. It is made available under aCC-BY 4.0 International license.

at any fixed time; $\bar{V}_G(t)$ depends on receptor information from previous times, due to the way in which G proteins interact with receptors. We obtain n approximately independent estimates for $\bar{V}_G$ by computing $\bar{V}_G$ at sufficiently spaced time points $t_1 < t_2 < \cdots < t_n$ where $t_{k+1} - t_k \geq 1s$.

To compare these direction estimates with the true gradient direction θ, we compute the angular deviation $\arccos(\bar{V} \cdot \theta)$ of the estimate $\bar{V}$ $(= \bar{V}_C, \bar{V}_{RL}, -\bar{V}_R, \bar{V}_G)$ from the true direction θ. The empirical cumulative distributions of these angular deviations are shown in (Fig. 1B, 1D, 2D, 3C) of the main text – this function of $\alpha \in [0, 180]$ is the fraction of the random estimates $\bar{V}$ within α degrees of the true direction θ. In (Fig. 2C, 3A, 3B) of the main text, we also show the fluctuation of these estimates over time, while the receptor and G protein states change asynchronously (but receptor positions are fixed).

1. Cramér-Rao bound

The estimates $\bar{V}_C$, $\bar{V}_{RL}$ and $-\bar{V}_R$ are random variables; in fact, they are functions of the receptor data $\{(X_k, R_k)\}_{k=1}^{N_R}$ at a fixed time. As such, we examine their efficiency by comparing their variance to theoretical lower bounds provided by the Cramér-Rao theory. The present setting is complicated by the fact that the parameter space for the direction parameter θ is the unit sphere $\mathcal{S}_1$, which is not a linear space. Nevertheless, the variance of an estimator on the sphere can be bounded from below in terms of the inverse ${\mathcal{I}_R}^{-1}$ of the Fisher information matrix

$$\mathcal{I}_R = \mathbb{E}_\theta \left[(\text{grad}_\theta \log \rho(Z, \theta))(\text{grad}_\theta \log \rho(Z, \theta))^T \right], \tag{B5}$$

where $\rho(Z, \theta)$ is given by (B1) with $Z = \{X_k, R_k\}_{k=1}^{N_R}$, and $\mathbb{E}_\theta$ denotes integration with respect to density $\rho(\cdot, \theta)$, parameterized by θ. This matrix $\mathcal{I}$ should be regarded as a symmetric 2×2 matrix acting on the tangent space at $\theta \in \mathcal{S}_1$. An applicable Cramér-Rao type bound is given in [37]. (See also Chapter 13 of [50]). It follows from Theorem 3.2 and Example 4.2 of [37] that the estimate $\bar{V}$ must satisfy

$$NSR = \frac{\mathbb{E}_\theta \left[\|\theta^\perp \bar{V}\|^2 \right]}{\|\mathbb{E}_\theta \left[\bar{V} \right]\|^2} \geq \text{trace}({\mathcal{I}_R}^{-1}) \tag{B6}$$

where $\theta^\perp Y = (I - \theta\theta^T)Y$ is the projection of vector $Y \in \mathbb{R}^3$ onto the subspace orthogonal to the vector θ. By definition $\bar{V}$ is normalized so that $\|\bar{V}\| = 1$; however, $\|\mathbb{E}_\theta[\bar{V}]\| < 1$. The

bioRxiv preprint doi: https://doi.org/10.1101/2025.04.18.649595; this version posted February 18, 2026. The copyright holder for this preprint (which was not certified by peer review) is the author/funder, who has granted bioRxiv a license to display the preprint in perpetuity. It is made available under aCC-BY 4.0 International license.

law of large numbers implies that as $N_R \to \infty$, $\mathbb{E}_\theta[\bar{V}] \to \theta$ and $\|\mathbb{E}_\theta[\bar{V}]\| \to 1$. For a weak signal and with few receptors, however, $\|\mathbb{E}_\theta[\bar{V}]\|$ may be small. Nevertheless, (B6) holds as long as the estimator $\bar{V}$ is unbiased and $\|\mathbb{E}_\theta[\bar{V}]\| \neq 0$, which is the case here for each of the estimators $\bar{V} = \bar{V}_C, \bar{V}_{RL}, \bar{V}_R$, by the symmetry of our choice of $C(x,\theta)$. In particular, $\|\mathbb{E}_\theta\left[\bar{V}\right]\| = |\mathbb{E}_\theta\left[\bar{V}\cdot\theta\right]|$ holds in our setting. In the ratio appearing in (B6), $\|\theta^\perp \bar{V}\|^2$ is zero if and only if $\bar{V}$ lies in the line spanned by the true direction θ; on the other hand, $\|\theta^\perp \bar{V}\|^2$ is maximal when $\bar{V}$ is orthogonal to θ. We also note that $\|\theta^\perp \bar{V}\|^2 = \sin^2(\alpha)$ where $\alpha = \arccos(\bar{V}\cdot\theta)$ is the angular deviation between $\bar{V}$ and θ.

We refer to the ratio in (B6) as the "noise to signal" ratio (NSR) for the estimate V. It is similar to a squared coefficient of variation $(CV)^2$ for the normalized vector $\bar{V}$: the numerator is the variance of the normalized vector $\bar{V}$ in the directions orthogonal to θ, while the denominator is the mean of $\bar{V}$ squared. On the other hand, this ratio differs from the usual notion of coefficient of variation in that the quantity $\bar{V}$ is vector-valued, and the denominator $\mathbb{E}_\theta\left[\bar{V}\right]$ is not the mean of $\theta^\perp \bar{V}$. Another Cramér-Rao type bound is given by [51] (see Theorem 2 and Corollary 2):

$$\mathbb{E}_\theta\left[d^2(\bar{V}\ ,\ \theta)\right] \geq \mathrm{trace}({\mathcal{I}_R}^{-1}) + \text{curvature terms}.$$

Here $d(u,v) = \arccos(u\cdot v)$ is the standard Riemannian distance between points u and v on the unit sphere, and the curvature terms (due to the positive curvature of $\mathcal{S}_1$) are negligible when $\mathbb{E}_\theta\left[d^2(\bar{V}\ ,\ \theta)\right]$ is small (when N_R is sufficiently large).

The Fisher information matrix can be computed, using the fact that the joint density ρ in (B1) has a product structure (the pairs $\{(X_k, R_k)\}$ are independent and identically distributed). One finds that

$$\mathcal{I}_R = \frac{N_R}{4\pi L^2}\int_{\mathcal{S}}(1-p_R(x,\theta))p_R(x,\theta)(\mathrm{grad}_\theta \log C(x,\theta))(\mathrm{grad}_\theta \log C(x,\theta))^T\,dx \tag{B7}$$

$$= \frac{N_R}{4\pi L^2}\int_{\mathcal{S}}\frac{(\mathrm{grad}_\theta p_R(x,\theta))(\mathrm{grad}_\theta p_R(x,\theta))^T}{(1-p_R(x,\theta))p_R(x,\theta)}\,dx \tag{B8}$$

Assuming p_R is a function of $x\cdot\theta$, then this symmetry of p_R about θ implies that $\mathcal{I}_R$ is a multiple of the 2×2 identity matrix. For various choices of $C(x,\theta)$, we evaluate the integral in (B8) numerically, and then compute $\mathrm{trace}({\mathcal{I}_R}^{-1})$ appearing in (B6). For $\bar{V} = \bar{V}_C, \bar{V}_{RL}, -\bar{V}_R$, we approximate the ratio

$$NSR = \frac{\mathbb{E}_\theta\left[\|\theta^\perp \bar{V}\|^2\right]}{\|\mathbb{E}_\theta\left[\bar{V}\right]\|^2} \tag{B9}$$

bioRxiv preprint doi: https://doi.org/10.1101/2025.04.18.649595; this version posted February 18, 2026. The copyright holder for this preprint (which was not certified by peer review) is the author/funder, who has granted bioRxiv a license to display the preprint in perpetuity. It is made available under aCC-BY 4.0 International license.

and compare with the lower bound (B6). The expectations are approximated by generating independent samples of $\bar{V}$ (as described above) and averaging over these samples. The comparisons are shown in (Fig. 1C) of the main text for different total receptor numbers N_R.

The G protein configuration at time t is described by pairs $\{Y_j(t), G_j(t)\}_{j=1}^{N_G}$. These variables and the corresponding direction estimate $\bar{V}_G$ are not a function of the receptor data at any fixed time: they depend on the history of the receptors up to time t. Nevertheless, we may ask how the variance of the direction estimate $\bar{V}_G$ compares to the estimates $\bar{V}_C$, $\bar{V}_{RL}$ and $-\bar{V}_R$, based on the receptor data at a fixed time. To make this comparison, we compute the ratio

$$\frac{\mathbb{E}_\theta\left[\|\theta^\perp \bar{V}_G\|^2\right]}{\|\mathbb{E}_\theta\left[\bar{V}_G\right]\|^2}, \tag{B10}$$

approximating the expectation by averaging over approximately independent samples of $\bar{V}_G$. This ratio is shown in (Fig. 2E, 3D, 4A, 4E) of the main text along with the same statistic for the direction estimates $\bar{V}_C$, $\bar{V}_{RL}$ and $-\bar{V}_R$. We see that when N_R is small ($N_R = 375$) and when using the ratiometric model for G protein deactivation, the ratio (B10) is below the lower bound trace($\mathcal{I}_R{}^{-1}$) in (B6), below the theoretical lower bound for the estimates $\bar{V}_C$, $\bar{V}_{RL}$, and $-\bar{V}_R$ which use receptor data at a fixed time only. This is because $\bar{V}_G$ is more concentrated near the true direction θ than are the other estimates, even more concentrated than what the theoretical limit allows for any estimator based only on receptor data at a fixed time. The estimator $\bar{V}_G$ is not a function of the receptor data at any fixed time, so the theoretical bound in (B6) does not apply to the variance ratio (B10) for $\bar{V} = \bar{V}_G$. We propose that the fact that the active G protein estimator violates this bound indicates that the G protein configuration is effectively averaging receptor data over a period of time, incorporating an effectively larger amount of receptor information than what is available at a fixed time.

2. Weak signal behavior of Fisher information

Let us suppose $C(x, \theta)$ is given by (A3) with $b \ll 1$ small. Then $\text{grad}_\theta \log C \approx b\theta^\perp \bar{x}$ where $\bar{x} = x/|x| = x/L$. In this case, the Fisher information $\mathcal{I}_R$ is close to a multiple of the

bioRxiv preprint doi: https://doi.org/10.1101/2025.04.18.649595; this version posted February 18, 2026. The copyright holder for this preprint (which was not certified by peer review) is the author/funder, who has granted bioRxiv a license to display the preprint in perpetuity. It is made available under aCC-BY 4.0 International license.

2x2 identity matrix, $\mathcal{I}_R \approx N_R\kappa^2 I$, with

$$\kappa^2 = \frac{b^2}{2|4\pi L^2|}\int_{\mathcal{S}} \|\theta^{\perp}\bar{x}\|^2(1-p_R(x,\theta))p_R(x,\theta)\,dx. \tag{B11}$$

When b is small, then $p_R(y,\theta) \approx C_0/(C_0+K_d)$. Hence,

$$\kappa^2 \approx b^2\frac{C_0K_d}{(C_0+K_d)^2}\frac{1}{2|\mathcal{S}_1|}\int_{\mathcal{S}_1}\|\theta^{\perp}\cdot y\|^2\,dy\,,$$

where $\mathcal{S}_1$ is the unit sphere. By symmetry, we have $\int_{\mathcal{S}_1}\|\theta^{\perp}\cdot y\|^2\,dy = \int_{\mathcal{S}_1}(y_1)^2+(y_2)^2\,dy = \frac{2}{3}|\mathcal{S}_1|$. Thus, we have

$$\text{trace}(\mathcal{I}_R{}^{-1}) \approx 2(N_R\kappa^2)^{-1} \approx \frac{6}{N_Rb^2}\frac{(C_0+K_d)^2}{K_dC_0}. \tag{B12}$$

This has the same form as the analogous expression in the two-dimensional model of [32] (c.f. equation (6) for σ_ϕ^2 therein, with $p = 2b$). In particular, as observed there, this quantity is minimized when $C_0 = K_d$, suggesting that sensing is most effective when $C_0 = K_d$.

Notice that the expression in (B12) diverges as $N_Rb^2 \to 0$, while the the numerator in (B6) always satisfies $\mathbb{E}_\theta\left[\|\theta^{\perp}\bar{V}\|^2\right] \leq 1$. Thus, it must also be true that the denominator in (B6) satisfies $\|\mathbb{E}_\theta\left[\bar{V}\right]\|^2 \leq O(N_Rb^2)$ as $b \to 0$. (This can be justified directly, by approximating the distribution of V (unnormalized) by a Gaussian with mean of $b\theta$ and covariance $N_R^{-1}I_{3\times3}$.) On the other hand, as $Nb^2 \to \infty$, $\|\mathbb{E}_\theta\left[\bar{V}\right]\| \to 1$. So, (B12) and (B6) together imply that when b is small and $N_Rb^2 \to \infty$,

$$\mathbb{E}_\theta\left[\|\theta^{\perp}\bar{V}\|^2\right] \geq \frac{6}{N_Rb^2}\frac{(C_0+K_d)^2}{K_dC_0}(1+o(1)). \tag{B13}$$

In particular, the mean squared angular deviation of $\bar{V}$ is of the order $1/(N_Rb^2)$. Nevertheless, the bound (B6) holds even when N_Rb^2 is not large.

3. Covariance and reliability thresholds

How large must N_R be in order that V_{RL}, $-V_R$, or V_C be a reliable estimate of the direction θ? The Cramér-Rao bound shows that the mean squared angular deviation of these estimates must be at least of order $N_R^{-1}b^{-2}$. Here we consider an upper bound on angular deviation of these estimates, and we derive an explicit threshold on N_R such that when N_R is above this threshold, the median angle deviation of these estimates will be

bioRxiv preprint doi: https://doi.org/10.1101/2025.04.18.649595; this version posted February 18, 2026. The copyright holder for this preprint (which was not certified by peer review) is the author/funder, who has granted bioRxiv a license to display the preprint in perpetuity. It is made available under aCC-BY 4.0 International license.

within a certain tolerance. As we will show, this framework also extends the estimate V_G based on G protein, and it is a useful perspective for comparing V_{RL}, $-V_R$, and V_C with V_G.

Given the true direction $\theta \in S$ of the ligand gradient, and given a tolerance $\delta > 0$, we say that a vector V (either $V = V_{RL}$, $-V_R$, or V_C) is a **δ-reliable** direction estimate for θ if

$$\mathbb{P}\left(\arccos\left(\frac{V}{|V|}\cdot\theta\right) \leq \delta\right) \geq \frac{1}{2}. \tag{B14}$$

Note that $\arccos(\frac{V}{|V|}\cdot\theta)$ is the angle between the vector θ and the vector V, even if V is not a unit vector. So, condition (B14) is equivalent to saying that the median angle deviation of V from θ is less than δ. Define the cone of angle δ around θ:

$$\mathcal{C}_{\delta,\theta} = \left\{x \in \mathbb{R}^3 \mid \; \arccos\left(\frac{x}{\|x\|},\theta\right) \leq \delta\right\}. \tag{B15}$$

Since the cone $\mathcal{C}_{\delta,\theta}$ is invariant under scaling $x \mapsto rx$ for any $r > 0$, $V \in \mathcal{C}_{\delta,\theta}$ if and only if $\bar{V} = V/|V| \in \mathcal{C}_{\delta,\theta}$. Thus, the reliability condition (B14) is equivalent to

$$\mathbb{P}\left(V \in \mathcal{C}_{\delta,\theta}\right) \geq \frac{1}{2}. \tag{B16}$$

For a fixed deviation tolerance (e.g. $\delta = 30°$), how large must N_R be in order that the estimates V_{RL}, $-V_R$, or V_C be reliable in this sense? While the exact distributions of V_{RL}, $-V_R$, and V_C are not explicit, we can estimate $\mathbb{P}\left(V \in \mathcal{C}_{\delta,\theta}\right)$ in terms of the means and covariances of $V = V_{RL}, -V_R, V_C$; this will provide a sufficient condition for δ-reliability. We say that a random vector V is unbiased if its mean $\mu = \mathbb{E}[V]$ is aligned with θ, which means that $\mu = |\mu|\theta$.

Proposition B.1 *Suppose that a random vector V has mean $\mu = \mathbb{E}[V]$ and covariance matrix $\Sigma = Cov(V)$. Suppose that V is unbiased: $\mu = |\mu|\theta$. If*

$$trace(\Sigma) \leq |\mu|^2 \sin^2(\delta)/2, \tag{B17}$$

then V is δ-reliable estimate of θ.

Proof: If $r \leq |\mu|\sin(\delta)$, then the ball of radius r centered at μ is contained in the cone $\mathcal{C}_{\delta,\theta}$. See Figure 8. In other words, the condition

$$|V - \mu| < |\mu|\sin(\delta)$$

bioRxiv preprint doi: https://doi.org/10.1101/2025.04.18.649595; this version posted February 18, 2026. The copyright holder for this preprint (which was not certified by peer review) is the author/funder, who has granted bioRxiv a license to display the preprint in perpetuity. It is made available under aCC-BY 4.0 International license.

implies $V \in \mathcal{C}_{\delta,\theta}$. By Chebyshev's inequality:

$$\mathbb{P}(|V-\mu| \geq |\mu|\sin(\delta)) \leq \frac{\mathbb{E}[|V-\mu|^2]}{|\mu|^2\sin^2(\delta)} = \frac{\text{trace}(\Sigma)}{|\mu|^2\sin^2(\delta)}.$$

If (B17) holds, then the right side is less than 1/2. Hence

$$\mathbb{P}\left(V \in \mathcal{C}_{\delta,\theta}\right) \geq \mathbb{P}(|V-\mu| < |\mu|\sin(\delta)) = 1 - \mathbb{P}\left(|V-\mu| \geq |\mu|\sin(\delta)\right) \geq 1/2. \tag{B18}$$

Thus, V is δ-reliable.

The condition (B17) is a sufficient condition for V to be δ-reliable, but it is not a necessary condition. Nevertheless, condition (B17) is easier to verify than computing the probability in (B16), and it is not based on any approximation.

We now apply this to the particular cases $V = V_{RL}, -V_R, V_C$. Let μ_{RL} denote the mean of V_{RL}:

$$\mu_{RL} = \mathbb{E}[V_{RL}] = N_R\mu_1, \qquad \mu_1 = \mathbb{E}[R_1X_1] = \frac{1}{|S|}\int_S p_R(x)x\,dx.$$

The mean of $-V_R$ is the same, since $\mathbb{E}[X_i] = 0$:

$$\mu_R = \mathbb{E}[-V_R] = N_R\mathbb{E}[(R_1-1)X_1] = N_R\mathbb{E}[R_1X_1] - N_R\mathbb{E}[X_1] = N_R\mu_1 = \mathbb{E}[V_{RL}].$$

Since the pairs $\{(X_k,R_k)\}_{k=1}^{N_R}$ are independent, the covariance matrix of V_{RL} is

$$\Sigma_{RL} = \text{Cov}(V_{RL}) = N_R\Sigma_{RL}^1,$$

where Σ_{RL}^1 is the covariance of R_1X_1:

$$\Sigma_{RL}^1 = \text{Cov}(R_1X_1) = \frac{1}{|S|}\int_S p_R(x)xx^T\,dx - \mu_1(\mu_1)^T. \tag{B19}$$

Similarly, the covariance matrix of $-V_R$ is

$$\Sigma_R = \text{Cov}(-V_R) = N_R\Sigma_R^1,$$

where Σ_R^1 is the covariance of $(R_1-1)X_1$:

$$\Sigma_R^1 = \text{Cov}((R_1-1)X_1) = \frac{1}{|S|}\int_S (1-p_R(x))xx^T\,dx - \mu_1(\mu_1)^T. \tag{B20}$$

The mean of μ_C of V_C is exactly twice the mean of V_{RL}:

$$\mu_C = \mathbb{E}[V_C] = N_R\mathbb{E}[(2R_1-1)X_1] = \frac{N_R}{|S|}\int_S (2p_R(x)-1)x\,dx = N_R2\mu_1 = 2\mu_{RL}.$$

bioRxiv preprint doi: https://doi.org/10.1101/2025.04.18.649595; this version posted February 18, 2026. The copyright holder for this preprint (which was not certified by peer review) is the author/funder, who has granted bioRxiv a license to display the preprint in perpetuity. It is made available under aCC-BY 4.0 International license.

The covariance of V_C is

$$\Sigma_C = N_R \Sigma_C^1$$

where Σ_C^1 is the covariance of $(2R-1)X_1$:

$$\Sigma_C^1 = \mathrm{Cov}((2R_1 - 1)X_1) = \frac{1}{|S|}\int_S xx^T\, dx - 4\mu_1(\mu_1)^T. \tag{B21}$$

Notice that the relation

$$\Sigma_C^1 = \Sigma_{RL}^1 + \Sigma_R^1 - 2\mu_1\mu_1^T$$

always holds. The estimates V_{RL}, $-V_R$, and V_C are all unbiased if μ_1 is aligned with θ: $\mu_1 = |\mu_1|\theta$.

By applying Proposition B.1 with $V = V_{RL}, -V_R, V_C$ we obtain the following thresholds for δ-reliability:

Corollary B.1 *Suppose that the estimates V_{RL}, $-V_R$, and V_C are unbiased: $\mu_1 = |\mu_1|\theta$. The reliability condition* (B17) *for V_{RL} is*

$$trace(\Sigma_{RL}) \le |\mu_{RL}|^2 \sin^2(\delta)/2$$

or, equivalently,

$$N_R \ge \frac{2 \cdot trace(\Sigma_{RL}^1)}{(\mu_1 \cdot \theta)^2 \sin^2(\delta)}. \tag{B22}$$

The reliability condition (B17) *for $-V_R$ is*

$$trace(\Sigma_R) \le |\mu_R|^2 \sin^2(\delta)/2$$

or, equivalently,

$$N_R \ge \frac{2 \cdot trace(\Sigma_R^1)}{(\mu_1 \cdot \theta)^2 \sin^2(\delta)}. \tag{B23}$$

The reliability condition (B17) *for V_C is*

$$trace(\Sigma_C) \le |\mu_C|^2 \sin^2(\delta)/2$$

or, equivalently,

$$N_R \ge \frac{2 \cdot trace(\Sigma_C^1)}{(2\mu_1 \cdot \theta)^2 \sin^2(\delta)}. \tag{B24}$$

bioRxiv preprint doi: https://doi.org/10.1101/2025.04.18.649595; this version posted February 18, 2026. The copyright holder for this preprint (which was not certified by peer review) is the author/funder, who has granted bioRxiv a license to display the preprint in perpetuity. It is made available under aCC-BY 4.0 International license.

We may interpret the quantity $\mu_1 \cdot \theta$ to be the "signal strength"; this calculation shows that reliability of V_{RL}, $-V_R$ and V_C requires that N_R is of the order $(\mu_1 \cdot \theta)^{-2}$, which is large when the signal is weak (shallow ligand gradient). Suppose that the gradient is weak: $C(x) = C_0(1 + bx \cdot \theta)$ with $b > 0$ small (here we assume $L = 1$). Then $p_R(x)$ is close to linear in x:

$$p_R(x) = \frac{C(x)}{C(x) + K_D} = p_R(0) + bp_R(0)(1 - p_R(0))x \cdot \theta + O(b^2)$$

and thus

$$(\mu_1 \cdot \theta) \approx b\, p_R(0)(1 - p_R(0))c_1, \qquad c_1 = \frac{1}{|\mathcal{S}_1|}\int_{\mathcal{S}_1} (x_1)^2\, dx = \frac{1}{3}, \qquad \Sigma^1_{RL} \approx p_R(0)c_1 I_3, \qquad \Sigma^1_R \approx (1 - p_R(0))c_1 I$$

where I_3 is the 3×3 identity matrix. Hence, in this weak gradient regime, the sufficient condition (B22) for V_{RL} to be δ-reliable reduces to

$$N_R \geq \frac{2 \cdot \mathrm{trace}(\Sigma^1_{RL})}{(\mu_1 \cdot \theta)^2 \sin^2(\delta)} \approx \frac{p_R(0)}{b^2(p_R(0))^2(1 - p_R(0))^2} \cdot \frac{18}{\sin^2(\delta)}. \tag{B25}$$

The condition (B23) for $-V_R$ to be δ-reliable reduces to

$$N_R \geq \frac{2 \cdot \mathrm{trace}(\Sigma^1_{R})}{(\mu_1 \cdot \theta)^2 \sin^2(\delta)} \approx \frac{(1 - p_R(0))}{b^2(p_R(0)^2(1 - p_R(0))^2} \cdot \frac{18}{\sin^2(\delta)}. \tag{B26}$$

The condition (B24) for V_C to be δ-reliable reduces to

$$N_R \geq \frac{2 \cdot \mathrm{trace}(\Sigma^1_{C})}{(2\mu_1 \cdot \theta)^2 \sin^2(\delta)} \approx \frac{1}{b^2(p_R(0))^2(1 - p_R(0))^2} \cdot \frac{9}{2\sin^2(\delta)}. \tag{B27}$$

Comparing (B25), (B26), and (B27), we see that when $p_R(0) \in (1/4, 3/4)$, V_C has the lowest threshold for reliability. When $p_R(0) = 1/2$ (i.e. $C_0 = K_d$), the thresholds (B25) and (B26) for V_{RL} and $-V_R$ are the same, and the threshold (B27) for V_C is exactly 1/2 of (B25) or (B26). In this scenario, V_C is effectively using twice the data as V_{RL} or $-V_R$. For $p_R(0)$ outside of the range $(1/4, 3/4)$, V_C is not the most reliable of the three estimates. When $p_R(0) < 1/4$ (i.e. $C_0 < (1/3)K_d$), the reliability threshold for V_{RL} ((B25)) is lowest among the three; when $p_R(0) > 3/4$ (i.e. $C_0 > 3K_d$), the reliability threshold for $-V_R$ ((B25)) is lowest among the three. The intuition about why V_C would perform worse than V_{RL} when $p_R(0) < 1/4$ is that in this regime the majority of receptors are inactive and their conditional angular distribution is more uniform than the conditional distribution of active receptor. Indeed, the conditional density of a receptor's location, conditioned on it being inactive, is proportional to $1 - p_R(x) = K_d/(K_d + C(x))$, and this density is more uniform when C_0/K_d is small. So, when C_0/K_d is small, the estimate V_C is dominated by

bioRxiv preprint doi: https://doi.org/10.1101/2025.04.18.649595; this version posted February 18, 2026. The copyright holder for this preprint (which was not certified by peer review) is the author/funder, who has granted bioRxiv a license to display the preprint in perpetuity. It is made available under aCC-BY 4.0 International license.

the contribution from inactive receptors which are approximately uniformly distributed on the sphere.

Another approach to estimating the probability in (B16) would be to approximate V by a Gaussian random vector having the same mean and covariance (justified by the central limit theorem when N_R is large). If V has mean $|\mu|\theta$ and covariance matrix $\frac{\sigma^2}{N_R} I_{3\times 3}$, then under this approximation

$$\mathbb{P}\left(V \in \mathcal{C}_{\delta,\theta}\right) \approx \mathbb{P}\left(|\mu|\theta + \frac{\sigma}{\sqrt{N_R}} Z \in \mathcal{C}_{\delta,\theta}\right) = \mathbb{P}\left(\frac{\sqrt{N_R}|\mu|}{\sigma}\theta + Z \in \mathcal{C}_{\delta,\theta}\right)$$

where Z is a standard Gaussian vector in $\mathbb{R}^3$ with mean zero and covariance $I_{3\times 3}$. Thus, V is δ-reliable once $N_R > \frac{\sigma^2}{|\mu|^2} c_\delta^2$, where the constant $c_\delta > 0$ is the smallest value r such that $\mathbb{P}\left(r\theta + Z \in \mathcal{C}_{\delta,\theta}\right) \geq 1/2$. Applied to V_{RL}, $-V_R$, and V_C, this approach yields thresholds with the same scaling as (B25), (B26), (B27), but with the less explicit universal constant c_δ^2.

Appendix C: Time averaging

In the ratiometric model, the instantaneous G protein states (active/inactive) are derived from historical receptor states. Each G protein's state is determined by its most recent interaction with a receptor, either active or inactive (Figure 9). If $N_G > N_R$, this mechanism may allow the G protein states to represent more information than what is encoded by the receptor states at a single time. Here we explain this mechanism. Throughout this section we consider only the ratiometric model for G protein dynamics.

1. Sampling the state of a single receptor at multiple random times

For simplicity, we first consider the case of a single receptor, and n G proteins interacting with it, and we ignore the spatial aspect of the model for now. The state of a given G protein is determined by the state of the receptor at the time of the protein's most recent encounter with that receptor. If the times at which the n proteins most recently interacted with that receptor are $t_1, t_2, \ldots, t_n$ then the states of the G proteins represent n samples of the historical state of the (single) receptor. These historical states are correlated, however; the correlation is higher when the interaction times are close together. If the interaction times are far apart, then sampling the state of the receptor at n different times can be similar to sampling n independent receptors.

bioRxiv preprint doi: https://doi.org/10.1101/2025.04.18.649595; this version posted February 18, 2026. The copyright holder for this preprint (which was not certified by peer review) is the author/funder, who has granted bioRxiv a license to display the preprint in perpetuity. It is made available under aCC-BY 4.0 International license.

The receptor state is a two-state, continuous-time Markov process $R(t)$; the two states are the active state (1) and the inactive state (0). The transition rates for this chain are given by (A1), where $x \in \mathcal{S}$ is the fixed location of the receptor. For clarity in notation we write $\lambda_+ = k_{\text{on}} C(x)$ for the activation rate, $\lambda_- = k_{\text{off}}$ for the inactivation rate, and we define $\lambda = \lambda_+ + \lambda_-$. The stationary distribution for this process is $\nu = (\nu(0), \nu(1))$, with

$$\nu(0) = \frac{\lambda_-}{\lambda_+ + \lambda_-}, \qquad \nu(1) = \frac{\lambda_+}{\lambda_+ + \lambda_-}.$$

So, $\nu(0)$ and $\nu(1)$ are the steady-state probabilities of finding the receptor in states 0 or 1, respectively. Let $p_t(z, y)$ denote the transition probabilities: $p_t(z, y)$ is the probability that the state at time $t > 0$ is y, given that the state at time 0 is z, $y, z \in \{0, 1\}$. These probabilities satisfy the system

$$\frac{d}{dt} p_t(z,0) = -\lambda_+ p_t(z,0) + \lambda_- p_t(z,1)$$
$$\frac{d}{dt} p_t(z,1) = \lambda_+ p_t(z,0) - \lambda_- p_t(z,1)$$

for $x \in \{0, 1\}$. This has an explicit solution. We find that the transition probabilities are

$$\begin{aligned}
p_t(0,0) &= \nu(0)\left(1 - e^{-\lambda t}\right) + e^{-\lambda t}, \\
p_t(0,1) &= \nu(1)\left(1 - e^{-\lambda t}\right), \\
p_t(1,0) &= \nu(0)\left(1 - e^{-\lambda t}\right), \\
p_t(1,1) &= \nu(1)\left(1 - e^{-\lambda t}\right) + e^{-\lambda t}.
\end{aligned}$$

In particular, regardless of the initial state x, $p_t(z, y)$ converges exponentially fast to $\nu(y)$, at rate $\lambda = \lambda_+ + \lambda_-$. Thus, $\lambda^{-1} = (k_{\text{on}} C + k_{\text{off}})^{-1}$ is the time scale for "loss of memory" or mixing of the receptor state.

Now, suppose the receptor process R has achieved stationarity, and suppose there are n G proteins that read the state of this single receptor at random times $t_1, \ldots, t_n$ (unordered). Assume these times are independent of each other and of the receptor process. Suppose that for each k, t_k is exponentially distributed with mean $\tau = \mathbb{E}[t_k]$. We will use $G_i \in \{0, 1\}$ to denote the state of the ith G protein (i.e. $G_i = R(t_i)$). Although the times $\{t_k\}_{k=1}^n$ are independent, the random variables $(G_1, \ldots, G_n) = (R(t_1), \ldots, R(t_n))$ are dependent, since the data come from the same receptor. However, the level of dependence is controlled by the dimensionless parameter $\lambda\tau$, as we now show. Let A_G denote the fraction of receptor

bioRxiv preprint doi: https://doi.org/10.1101/2025.04.18.649595; this version posted February 18, 2026. The copyright holder for this preprint (which was not certified by peer review) is the author/funder, who has granted bioRxiv a license to display the preprint in perpetuity. It is made available under aCC-BY 4.0 International license.

readings that are in the active state:

$$A_G = \frac{1}{n}\sum_{i=1}^{n} \mathbb{I}_{G_i=1} = \frac{1}{n}\sum_{k=1}^{n} R(t_k).$$

Let $p_R = \nu(1) = \frac{\lambda_+}{\lambda_+ + \lambda_-} = \mathbb{P}(R(t) = 1)$ be the probability that the receptor is active (R is statistically stationary, so this probability does not change with time). The autocorrelation function for the receptor state is: $\mathbb{E}[R(t)R(t+s)] = p_R^2 + p_R(1-p_R)e^{-\lambda s}$.

Proposition C.1 *The mean and variance of A_G are $\mathbb{E}[A_G] = p_R$ and*

$$Var(A_G) = \frac{1}{n}p_R(1-p_R) + \left(1 - \frac{1}{n}\right)\frac{p_R(1-p_R)}{(1+\lambda\tau)}. \tag{C1}$$

The first term in this expression, $p_R(1-p_R)/n$, would be the variance if the n readings were independent (e.g. taken separately from n independent receptors); in the limit $n \to \infty$ this term vanishes. The second term in the variance is the result of temporal correlation in the receptor process. As $\lambda\tau \to \infty$, the last term vanishes, leaving only $p_R(1-p_R)/n$.

2. Effective receptor number

Let us compare this result to an alternative model, which will motivate the notion of **effective receptor number**. Instead of there being a single receptor with state changing in time, let us now suppose there is a cluster of n_R receptors with independent states that do not change in time. Let p_R be the probability that a given receptor in the cluster is active. Suppose that each of the n G proteins samples a state from this cluster: each protein chooses one of the n_R receptors at random and records its state. So, two proteins that choose the same receptor must record the same state. As before, let A_G be the fraction of readings that are in the active state:

$$A_G = \frac{1}{n}\sum_{i=1}^{n} \mathbb{I}_{G_i=1}.$$

Proposition C.2 *The mean and variance of A_G are $\mathbb{E}[A_G] = p_R$ and*

$$Var(A_G) = \frac{1}{n}p_R(1-p_R) + \left(1 - \frac{1}{n}\right)\frac{p_R(1-p_R)}{n_R}. \tag{C2}$$

Both models have the same mean p_R for all parameter choices. Comparing (C1) and (C2), we see that the variance in the two models is the same when $(1+\lambda\tau) = n_R$. Thus, we

bioRxiv preprint doi: https://doi.org/10.1101/2025.04.18.649595; this version posted February 18, 2026. The copyright holder for this preprint (which was not certified by peer review) is the author/funder, who has granted bioRxiv a license to display the preprint in perpetuity. It is made available under aCC-BY 4.0 International license.

could think of $(1+\lambda\tau)$ as an **effective number of receptors**: when the G proteins read a single receptor at independent random times, it is as if each G protein reads a state chosen randomly and independently from a group of $(1+\lambda\tau)$ independent receptors. The fact that the G proteins read historical states effectively expands the pool of independent receptors. Interestingly, the quantity $n_R = (1+\lambda\tau)$ does not depend on n, assuming $n > 1$.

3. Derivation of Proposition C.1 and Proposition C.2

For both models, the variance of A_G is

$$\begin{aligned}\mathrm{Var}(A_G) &= \sum_{i=1}^{n} \mathrm{Var}(\mathbb{I}_{G_i=1}) + \sum_{i\neq k} \mathrm{Cov}(\mathbb{I}_{G_i=1}, \mathbb{I}_{G_k=1}) \\ &= np_R(1-p_R) + (n^2-n)\mathrm{Cov}(\mathbb{I}_{G_1=1}, \mathbb{I}_{G_2=1}), \end{aligned} \tag{C3}$$

and the covariance term is

$$\mathrm{Cov}(\mathbb{I}_{G_1=1}, \mathbb{I}_{G_2=1}) = \mathbb{P}(G_1=1, G_2=1) - \mathbb{P}(G_1=1)\mathbb{P}(G_2=1) \tag{C4}$$

$$= \mathbb{P}(G_1=1, G_2=1) - p_R^2. \tag{C5}$$

For the model considred in Proposition C.1, the joint density of the independent times (t_1, t_2) is $\rho(t_1,t_2) = \alpha^2 e^{-\alpha(t_1+t_2)}$ for $t_1, t_2 \geq 0$. Therefore,

$$\begin{aligned}\mathbb{P}(G_1=1, G_2=1) &= \int_0^\infty\int_0^\infty \mathbb{P}(R(t_1)=1, \quad R(t_2)=1)\rho(t_1,t_2)\,dt_1\,dt_2 \\ &= 2\iint_{t_1\leq t_2} \mathbb{P}(R(t_2)=1 \mid R(t_1)=1)\mathbb{P}R(t_1)=1)\alpha^2 e^{-\alpha(t_1+t_2)}\,dt_1\,dt_2 \\ &= 2p_R\iint_{t_1\leq t_2} \left(e^{-\lambda(t_2-t_1)} + p_R(1-e^{-\lambda(t_2-t_1)})\right)\alpha^2 e^{-\alpha(t_1+t_2)}\,dt_1\,dt_2 \\ &= p_R^2 + 2p_R(1-p_R)\iint_{t_1\leq t_2} e^{-\lambda(t_2-t_1)}\alpha^2 e^{-\alpha(t_1+t_2)}\,dt_1\,dt_2 \\ &= p_R^2 + p_R(1-p_R)\left(\frac{\alpha}{\lambda+\alpha}\right). \end{aligned} \tag{C6}$$

Therefore,

$$\mathrm{Cov}(\mathbb{I}_{G_1=1}, \mathbb{I}_{G_2=1}) = p_R(1-p_R)\left(\frac{1}{(\lambda/\alpha)+1}\right),$$

and

$$\mathrm{Var}(A_G) = np_R(1-p_R) + \left(n^2-n\right)p_R(1-p_R)\left(\frac{1}{(\lambda/\alpha)+1}\right). \tag{C7}$$

bioRxiv preprint doi: https://doi.org/10.1101/2025.04.18.649595; this version posted February 18, 2026. The copyright holder for this preprint (which was not certified by peer review) is the author/funder, who has granted bioRxiv a license to display the preprint in perpetuity. It is made available under aCC-BY 4.0 International license.

For the model considered in Proposition C.2,

$$\begin{aligned}\mathbb{P}(G_1 = 1, G_2 = 1) &= p_R \mathbb{P}(\text{two proteins choose the same receptor}) \\ &\quad + p_R^2 \mathbb{P}(\text{two proteins choose different receptors}) \\ &= p_R \frac{1}{n_R} + p_R^2 \left(1 - \frac{1}{n_R}\right). \end{aligned} \tag{C8}$$

Therefore,

$$\mathrm{Cov}(\mathbb{I}_{G_1=1}, \mathbb{I}_{G_2=1}) = \frac{p_R(1-p_R)}{n_R}.$$

Appendix D: Spatially extended, ratiometric model

In the analysis above we considered a population of G proteins interacting with a single receptor. Here we show how these ideas extend to the case when there are multiple receptors distributed spatially across the cell surface, and the G proteins interact with them, according to the ratiometric model, as the proteins diffuse over the surface. Throughout this section, we assume the ratiometric model, so $V_G = V_G^r$.

1. G protein ages

Considering the times at which a G protein interacts with a receptor, we define the **age** of a G protein at time t to be $s \geq 0$ if the last interaction between that G protein and some receptor occured at time $t - s$. If the G protein has age s, then that G protein has been in the same state for at least time $t - s$. Here we say "at least" time $t - s$ because it could be that the G protein was already in that state just before this most recent encounter with a receptor. For example, when an already-active G protein encounters an active receptor, the G protein's age will reset to zero but its state does not change. (See Figure 9). We will say that a G protein at time t is **linked** to a particular receptor if that G protein's last interaction was with that particular receptor – if that G protein's state at time t was recorded from that particular receptor (at time $t - s$, where s is the protein's age). Each G protein is linked to only one receptor at a time. Two or more G proteins may be linked to the same receptor, which creates dependence between those G protein states. (See Figure 10). In fact, this linking is the only source of statistical dependence between G protein states: G protein states are dependent only through interaction with the same receptor. Conditioned

bioRxiv preprint doi: https://doi.org/10.1101/2025.04.18.649595; this version posted February 18, 2026. The copyright holder for this preprint (which was not certified by peer review) is the author/funder, who has granted bioRxiv a license to display the preprint in perpetuity. It is made available under aCC-BY 4.0 International license.

on being linked to two different receptors, two G protein states are statistically independent. However, for G proteins linked to the same receptor, their ages may be different, and the observations above suggest that if these ages are sufficiently far apart, then the recorded states are approximately independent. As explained above in Proposition C.1, the level of dependence/independence between proteins linked to the same receptor is controlled by the ratio of two times scales – the mean G protein age and the receptor turnover time.

We can estimate the typical age of a G protein. If there are a total of N_R receptors, then the typical distance between receptors is of the order $\delta = \sqrt{(4\pi L^2/N_R)}$ where L is the cell radius. This is because $4\pi L^2/N_R$ is the surface area per receptor, and the surface is two-dimensional (hence, the square root). Therefore, a G protein diffusing on the cell surface is navigating among receptors that are typically distance δ apart. If we want to know when a given G protein last interacted with some receptor (any receptor), it is the same as looking *backwards* in time and asking when does a Brownian motion first hit an r_*-neighborhood of some receptor, among the N_R receptors distributed across the surface approximately δ apart. If the receptors have an interaction radius of $r_* \ll \delta$ and the G protein diffusion coefficient is D_G, then the typical **age** of a G protein will be of the order

$$\tau = \frac{\delta^2}{D_G} \ln(\delta/r_*) = \frac{L^2}{N_R D_G} \ln(\delta/r_*). \tag{D1}$$

We may also describe this quantity as the time scale of diffusive encounters between a G protein and receptors. This is motivated by thinking about a periodic array of discs in $\mathbb{R}^2$, having radius r_* and with centers δ apart. The quantity $\frac{\delta^2}{D_G} \ln(\delta/r_*)$ is the typical time it takes for a Brownian motion (with diffusion coefficient D_G) to hit one of the discs (any disc), when started from a random location. (The logarithm is an artifact of the 2-dimensional nature of the cell surface and of the Green's function in 2-dimensions.) Here and elsewhere we drop constants of proportionality, such as 4π, that do not depend on the parameters, since we are mainly interested in the scaling with parameters. This time τ is not the typical time required for a given G protein to find a *particular* receptor. Rather, it is the time required (looking backward in time) to encounter any one of the receptors – this later quantity is what determines the age of a G protein. The relation in (D1) can be derived as in Appendix B of [28]; ignoring constants of proportionality that do not depend on the parameters, (D1) above has the same form as Eq. 14 from [28]. See also [52], [53], and references therein for a broader discussion of first-passage time calculations. This suggests that the age of a G

bioRxiv preprint doi: https://doi.org/10.1101/2025.04.18.649595; this version posted February 18, 2026. The copyright holder for this preprint (which was not certified by peer review) is the author/funder, who has granted bioRxiv a license to display the preprint in perpetuity. It is made available under aCC-BY 4.0 International license.

protein is a random variable that is approximately exponentially distributed with mean τ. If the number of G proteins is $N_G > N_R$, then on average there are (N_G/N_R) G proteins per receptor. So, if the receptors and proteins are uniformly distributed on the sphere, we should expect that for each of the N_R receptors there are about (N_G/N_R) G proteins that are **linked** with that receptor, in the sense defined above.

2. Covariance for G protein, fixed receptor locations

As we did with the simple single-receptor model above, we now consider how the time τ effects the variation of the G protein signal. Specifically, we show how for the ratiometric model the covariance of V_G depends on the time scale τ and the time scale of receptor dynamics. Here we suppose that the receptor locations are fixed at $X_1, \dots, X_{N_R}$. The receptor states are changing randomly, but their locations are fixed. A receptor at X_i activates at rate $k_{\rm on}C(X_i)$ and deactivates at rate $k_{\rm off}$; thus, the time scale for receptor dynamics at location X_i is $\lambda^{-1}(X_i) = (k_{\rm on}C(X_i) + k_{\rm off})^{-1}$. Suppose there are N_G proteins that are placed at locations $\{Y_j\}_{j=1}^{N_G}$, which are independent and uniformly distributed on the sphere. We consider their states $\{G_j\}_{j=1}^{N_G}$ at a fixed time, where $G_j \in \{0, 1\}$. We are interested in the vector

$$V_G = \sum_{j=1}^{N_G} G_j Y_j$$

which is the sum of the positions of active G proteins. Let $\mu_G = \mathbb{E}[V_G]$ be the mean of V_G, and let $\Sigma_G = \mathrm{Cov}(V_G)$ be the covariance matrix. As in (B16), we say that the G protein signal V_G is a **δ-reliable** estimate if

$$\mathbb{P}\left(V_G \in \mathcal{C}_{\delta,\theta}\right) \geq \frac{1}{2}. \tag{D2}$$

Proposition B.1 applied with $V = V_G$ gives us a sufficient condition for δ-reliability of V_G:

Corollary D.1 *Suppose that the estimate V_G is unbiased. If trace$(\Sigma_G) \leq |\mu_G|^2 \sin^2(\delta)/2$, then V_G is δ-reliable.*

To make use of this criteria and to compare the reliability of V_G with the reliability of V_{RL}, we need to compute μ_G and Σ_G. We now explain how these can be calculated for the ratiometric model.

bioRxiv preprint doi: https://doi.org/10.1101/2025.04.18.649595; this version posted February 18, 2026. The copyright holder for this preprint (which was not certified by peer review) is the author/funder, who has granted bioRxiv a license to display the preprint in perpetuity. It is made available under aCC-BY 4.0 International license.

The state G_j of the protein at position Y_j was read from a particular receptor at some previous time – we say that this protein is **linked** to that particular receptor. Define random variables $\{L_j\}_{j=1}^{N_G}$ which indicate the receptor to which the jth protein is linked: $L_j = k$ means that the jth protein (at Y_j) is linked to the kth receptor (which is at position X_k). Possibly multiple G proteins can be linked to the same receptor, but they may or may not read the same state from that receptor (because they interacted with that receptor at different times, and the receptor state is changing over time).

Let $\eta_i(y)$ denote the conditional density of Y_j, given $L_j = i$. This η_i is depends on the geometric arrangement of all receptors $\{X_k\}_{k=1}^{N_R}$, and we denote this dependence by $\eta_i([X], y)$. Although this quantity is impossible to compute exactly/explicitly, we should think of this as being concentrated in a neighborhood of X_i, as G proteins are most likely to be linked to nearby receptors. Thus,

$$\mathbb{P}(Y_j \in A \mid L_j = i) = \int_A \eta_i([X], y)\, dy, \qquad A \subset S.$$

Given $L_j = i$, the jth G protein takes its state from the ith receptor at some time T_j. Motivated by the analysis above, we make the simplifying assumption that this time (the age of that protein) is exponentially distributed with mean τ, $T_j \sim \text{Exponential}(1/\tau)$, where the parameter τ is given by (D1). We also assume that the ages are independent of G protein positions.

Proposition D.1 *Assume the ratiometric model for G protein dynamics. Given fixed receptor locations $\{X_i\}_{i=1}^{N_R}$, the mean of V_G is*

$$\mu_G = \mathbb{E}[V_G] = N_G \sum_{i=1}^{N_R} p_R(X_i) \int_S \eta_i([X], y) y\, dy \mathbb{P}(L_1 = i).$$

The covariance matrix $\Sigma_G = \mathbb{E}[(V_G - \langle V_G \rangle)(V_G - \langle V_G \rangle)^T]$ of V_G is

$$\Sigma_G = N_G M_1 + \frac{(N_G^2 - N_G)}{N_R} M_2 \tag{D3}$$

bioRxiv preprint doi: https://doi.org/10.1101/2025.04.18.649595; this version posted February 18, 2026. The copyright holder for this preprint (which was not certified by peer review) is the author/funder, who has granted bioRxiv a license to display the preprint in perpetuity. It is made available under aCC-BY 4.0 International license.

where

$$M_1 = \sum_{i=1}^{N_R} p_R(X_i) \int_S \eta_i([X], y) y y^T \, dy \mathbb{P}(L_1 = i)$$
$$- \sum_{m=1}^{N_R} \sum_{i=1}^{N_R} p_R(X_m) p_R(X_i) \int_S \int_S \eta_m([X], z) \eta_i([X], y) y z^T \, dy \, dz \mathbb{P}(L_1 = i) \mathbb{P}(L_2 = m) \tag{D4}$$

(D5)

and

$$M_2 = N_R \sum_{i=1}^{N_R} \frac{p_R(X_i)(1 - p_R(X_i))}{1 + \tau\lambda(X_i)} \int_S \int_S \eta_i([X], z) \eta_i([X], y) y z^T \, dy \, dz \mathbb{P}(L_1 = L_2 = i). \tag{D6}$$

The quantity

$$\int_S \eta_i([X], y) y \, dy$$

in the expression for μ_G is the mean location of a G protein linked to the ith receptor (at location X_i). Observe that the covariance matrix Σ_G is the sum of two terms. The second term, M_2, results from correlations caused by G proteins possibly interacting with the same receptor. This M_2 is the term where time-scales play a role, through $\tau\lambda(X_i)$. When $\tau\lambda$ is large, meaning that receptor dynamics are fast relative to G protein ages, then M_2 may be negligible.

From Proposition D.1 we can identify two parameter regimes corresponding to different behavior of the random fluctuation of V_G. The covariance of $\frac{1}{N_G} V_G$ is

$$\mathrm{Cov}(V_G/N_G) = (N_G)^{-2} \Sigma_G = \frac{1}{N_G} M_1 + \frac{(1 - 1/N_G)}{N_R} M_2.$$

Recall that the matrix M_2 depends on τ and λ. The quantity $\lambda(x) = k_{\mathrm{on}} C(x) + k_{\mathrm{off}}$ depends on x. Nevertheless, when the gradient is shallow, we have $\lambda \approx \lambda_0 = k_{\mathrm{on}} C_0 + k_{\mathrm{off}}$. Thus, Proposition D.1 shows that the dimensionless parameter $\lambda_0 \tau = (k_{\mathrm{on}} C_0 + k_{\mathrm{off}})\tau$, which is ratio of the G protein age τ with the receptor turnover time λ_0^{-1}, plays an important role. (The product $\lambda_0 \tau$ is analogous to a Damköhler number.) This analysis suggests two qualitatively different regimes for the behavior of the covariance of V_G:

- **The G-limited regime.** If

$$N_G \ll (1 + \lambda_0 \tau) N_R = N_R + \frac{\lambda_0 L^2}{D_G} \ln(\delta / r_*)$$

bioRxiv preprint doi: https://doi.org/10.1101/2025.04.18.649595; this version posted February 18, 2026. The copyright holder for this preprint (which was not certified by peer review) is the author/funder, who has granted bioRxiv a license to display the preprint in perpetuity. It is made available under aCC-BY 4.0 International license.

then we have

$$\text{Cov}(\frac{1}{N_G}V_G) \approx \frac{1}{N_G}M_1.$$

In this regime, the covariance is approximately that of N_G proteins that are sampling independent receptors. Either N_R is large or $\lambda_0\tau$ is large, so that the receptor states sampled by the G proteins are approximately independent. Consequently, increasing N_R or $\lambda_0\tau$ further does not decrease the covariance as significantly as would an increase in N_G. In other words, small N_G is the main limitation for reliability of V_G in this regime. Note: it is possible that $N_R < N_G < (1+\lambda_0\tau)N_R$. In other words, even if $N_R < N_G$, the time scales may be such that the covariance of V_G is relatively insensitive to changes in N_R because $(1+\lambda_0\tau)N_R \gg N_G$. This is consistent with our observation that the estimate V_G^r may be robust even at low N_R, as illustrated in Figure 2 and 3 of the main text. In particular, in this regime the noise-to-signal ratio for V_G^r may be smaller than trace$(\mathcal{I}_R^{-1})$ from (B6), which is the theoretical lower bound that applies to V_C and to any unbiased estimate based on instantaneous receptor states at a single fixed time.

- **The Receptor-limited regime.** If

$$N_G \gg (1+\lambda_0\tau)N_R = N_R + \frac{\lambda_0 L^2}{D_G}\ln(\delta/\epsilon),$$

then we have

$$\text{Cov}(\frac{1}{N_G}V_G) \approx \frac{1}{N_R}M_2.$$

In this regime, there are typically many G proteins linked to a given receptor. Variation of the estimate V_G is dominated by fluctuation in the receptor states (which are passed on to the G protein), and there is a relatively high level of correlation in the receptor states recorded by the G proteins linked to the same receptor. Increasing N_G further does not decrease the covariance as significantly as would an increase in either N_R or $\lambda_0\tau$. In other words, even though N_G is large, N_R is the main limitation for reliability of V_G in this regime.

In view of these calculations, we define the quantity

$$\tilde{N}_R = (1+\lambda_0\tau)N_R = N_R + \frac{\lambda_0 L^2}{D_G}\ln(\delta/\epsilon).$$

bioRxiv preprint doi: https://doi.org/10.1101/2025.04.18.649595; this version posted February 18, 2026. The copyright holder for this preprint (which was not certified by peer review) is the author/funder, who has granted bioRxiv a license to display the preprint in perpetuity. It is made available under aCC-BY 4.0 International license.

We call this the **effective receptor number** – this number represents an effective number of receptor states made available through historical sampling. The quantity $\tilde{N}_R$ should not be thought of as something that applies to a particular cell. Instead, $\tilde{N}_R$ can be regarded as a property of the ratiometric model, a model whose statistical properties are completely determined by parameters N_R, N_G, D_G, L, r_*, k_{off}, K_d and the ligand concentration $C(x)$.

Considering how these two regimes depend on the parameters, we make the following observations:

- Regarding D_G: Faster diffusion of G protein (D_G large) reduces τ. This is because when the diffusion of G protein is fast, the proteins typically interact with receptors very frequently, and their age is lower, so their states are more correlated with other G proteins that also hit the same receptor in the very recent past (the states of linked G proteins are likely to coincide). In the hypothetical extreme $D_G \to \infty$, the age of a G protein goes to zero so that the G proteins are recording only information from the present state of the receptors. In this case, $\tilde{N}_R \approx N_R$. Conversely, slower diffusion (D_G small) increases τ and the effective receptor number $\tilde{N}_R$. This is because with slow diffusion of G protein, their ages tend to be larger and further apart, so even the states of linked G proteins (i.e. G proteins that are linked to the same receptor) are approximately independent.

- Regarding λ_0: Faster receptor dynamics $\lambda_0 \gg 1$ increases $\tilde{N}_R$. This is because the states of a given receptor $R(t_1), \ldots, R(t_n)$ at some times $t_1 < t_2 < \cdots < t_n$ (that do not depend on λ_0) will be approximately independent when λ_0 is large. Thus, if multiple G proteins obtained their state by last interacting with that same receptor, then those proteins are encoding approximately independent state values. Conversely, slow receptor dynamics ($\lambda_0 \ll 1$) decreases $\tilde{N}_R$. A key point here is that changing the receptor turnover time λ_0^{-1} has no effect on the distribution of G protein ages.

- Regarding r_*: decreasing the interaction radius r_* will tend to increase G protein ages relative to the receptor turnover time, since it is more difficult for a protein to reach a receptor. This leads to greater independence in the states encoded by the G protein ensemble.

Thus far we have reasoned under the assumption that ligand concentration $C(x)$ is not

bioRxiv preprint doi: https://doi.org/10.1101/2025.04.18.649595; this version posted February 18, 2026. The copyright holder for this preprint (which was not certified by peer review) is the author/funder, who has granted bioRxiv a license to display the preprint in perpetuity. It is made available under aCC-BY 4.0 International license.

far from uniform:

$$\lambda(x) = k_{\text{on}}C(x) + k_{\text{off}} \approx \lambda_0 = k_{\text{on}}C_0 + k_{\text{off}} = k_{\text{off}}\left(C_0/K_d + 1\right).$$

With non-uniform ligand concentration, the receptor turnover time depends on position. So, in principle, the G protein on front end of the cell (where ligand concentration $C(\theta)$ is highest) should encode a higher level of independence than on the back end, because λ is larger on the front end. However, in the scenarios we are considering the ratio $(C(\theta) + K_d)/(C(-\theta) + K_d))$, with θ denoting the gradient direction, is not very large. So, the difference in this effect between front and back may not be significant.

3. Comparing V_G^r with V_{RL} when N_R is sufficiently large

These expressions for the mean μ_G and covariance Σ_G of V_G^r are complicated. In particular, explicitly computing the probability of linking to a particular receptor is not feasible, as it depends on the geometric configuration of all receptors. Nevertheless, we can gain further insight by making a simplifying assumption that N_R is large and the receptors are evenly distributed over S. This will allow us to simplify μ_G and Σ_G and compare them with μ_R and Σ_R, the mean and covariance of V_{RL}. In particular, we will show that in certain parameter regimes, V_G^r may be a more reliable estimate of θ than V_{RL}. This analysis is consistent with the results of our numerical simulations.

Under the assumption that N_R is large and the receptor locations are well-spaced over S, we expect that a G protein placed uniformly at random on S is equally likely to link with any receptor:

$$\mathbb{P}(L_1 = i) \approx 1/N_R, \quad \forall i = 1, \dots, N_R. \tag{D7}$$

Furthermore, under this assumption we expect that the conditional density $y \mapsto \eta_i([X], y)$ is concentrated in a small neighborhood of X_i. Thus, we approximate

$$\int_S \eta_i([X], y) y \, dy \approx X_i, \qquad \int_S \eta_i([X], y) y y^T \, dy \approx X_i X_i^T, \qquad \forall i = 1, \dots, N_R. \tag{D8}$$

Using the approximations (D7) and (D8) we have the following approximations of the mean and covariance of V_G^r. Recall from (B20), that V_{RL} satisfies

$$\mathbb{E}[V_{RL}] = N_R \mu_1, \qquad \text{Cov}(V_{RL}) = N_R \Sigma_{RL}^1,$$

bioRxiv preprint doi: https://doi.org/10.1101/2025.04.18.649595; this version posted February 18, 2026. The copyright holder for this preprint (which was not certified by peer review) is the author/funder, who has granted bioRxiv a license to display the preprint in perpetuity. It is made available under aCC-BY 4.0 International license.

where

$$\mu_1 = \int_S p_R(x)x\,dx, \qquad \Sigma^1_{RL} = \frac{1}{|S|}\int_S p_R(x)xx^T\,dx - \mu_1(\mu_1)^T.$$

Proposition D.2 *Using the approximations* (D7) *and* (D8) *and assuming N_R is large, the mean μ_G and covariance Σ_G of V^r_G are*

$$\mu_G = \mathbb{E}[V^r_G] \approx N_G\mu_1$$

and

$$\Sigma_G = Cov(V^r_G) \approx N_G\Sigma^1_{RL} + \frac{(N_G^2 - N_G)}{N_R}\Sigma_\tau,$$

where

$$\Sigma_\tau = \frac{1}{|S|}\int_S \frac{p_R(x)(1-p_R(x))}{1+\tau\lambda(x)}xx^T\,dx.$$

Using this, we can derive the following remarkable consequence about reliability of V^r_G in the G-limited regime:

Corollary D.2 *When $N_G > N_R$ and $\tau\lambda$ is large, the estimate V^r_G of θ may be δ-reliable, even while V_{RL} is not δ-reliable.*

Proof: Using these approximations for μ_G and Σ_G, the condition given in Corollary D.1 for δ-reliability of V^r_G is (approximately):

$$\frac{1}{N_G}\mathrm{trace}(\Sigma^1_{RL}) + \frac{(1-1/N_G)}{N_R}\mathrm{trace}(\Sigma_\tau) \le |\mu_1|\sin^2(\delta)/2.$$

In particular, this holds if

$$\frac{1}{N_G}\mathrm{trace}(\Sigma^1_{RL}) + \frac{1}{N_R}\mathrm{trace}(\Sigma_\tau) \le |\mu_1|\sin^2(\delta)/2. \qquad \text{(D9)}$$

On the other hand, the δ-reliability condition for V_{RL}, from Corollary B.1 is

$$\frac{1}{N_R}\mathrm{trace}(\Sigma^1_{RL}) \le |\mu_1|\sin^2(\delta)/2. \qquad \text{(D10)}$$

The matrix Σ_τ depends on $\tau\lambda$, but Σ^1_{RL} does not. For any N_R, we may take δ small enough so that V_{RL} is **not** δ-reliable. In particular, (D10) does not hold if δ is small. For this same value of N_R and δ, however, we may take $N_G > N_R$, and then $\lambda\tau$ large enough so that the inequality (D9) holds, implying that V^r_G is δ-reliable, even though V_{RL} is not. In particular, it would be necessary for $\mathrm{trace}(\Sigma_\tau) \le (1 - N_R/N_G)\mathrm{trace}(\Sigma^1_{RL})$.

bioRxiv preprint doi: https://doi.org/10.1101/2025.04.18.649595; this version posted February 18, 2026. The copyright holder for this preprint (which was not certified by peer review) is the author/funder, who has granted bioRxiv a license to display the preprint in perpetuity. It is made available under aCC-BY 4.0 International license.

916 ## 4. Derivation of Proposition D.1

The mean of V_G is:

$$\mathbb{E}[V_G] = \sum_{j=1}^{N_G} \mathbb{E}[G_j Y_j] = N_G \mathbb{E}[G_1 Y_1] = N_G \sum_{i=1}^{N_R} \mathbb{E}[G_1 Y_1 \mid L_1 = i] \mathbb{P}(L_1 = i)$$
$$= N_G \sum_{i=1}^{N_R} \mathbb{E}[R_1] \mathbb{E}[Y_1 \mid L_1 = i] \mathbb{P}(L_1 = i)$$
$$= N_G \sum_{i=1}^{N_R} p_R(X_i) \int_S \eta_i([X], y) y \, dy \mathbb{P}(L_1 = i). \quad \text{(D11)}$$

The covariance matrix $\Sigma_G = \mathbb{E}[(V_G - \langle V_G \rangle)(V_G - \langle V_G \rangle)^T]$ is:

$$\Sigma_G = N_G \mathbb{E}[(G_1 Y_1 - \langle G_1 Y_1 \rangle)(G_1 Y_1 - \langle G_1 Y_1 \rangle)^T]$$
$$+ (N_G^2 - N_G) \mathbb{E}[(G_1 Y_1 - \langle G_1 Y_1 \rangle)(G_2 Y_2 - \langle G_2 Y_2 \rangle)^T]. \quad \text{(D12)}$$

The first term is:

$$\mathbb{E}[(G_1 Y_1 - \langle G_1 Y_1 \rangle)(G_1 Y_1 - \langle G_1 Y_1 \rangle)^T] = \mathbb{E}[(G_1 Y_1)(G_1 Y_1)^T] - \mathbb{E}[(G_1 Y_1)] \mathbb{E}[(G_1 Y_1)^T] \quad \text{(D13)}$$

and

$$\mathbb{E}[(G_1 Y_1)(G_1 Y_1)^T] = \sum_{i=1}^{N_R} \mathbb{E}[(G_1 Y_1)(G_1 Y_1)^T \mid L_1 = i] \mathbb{P}(L_1 = i)$$
$$= \sum_{i=1}^{N_R} \mathbb{E}[R_i^2] \mathbb{E}[Y_1 Y_1^T \mid L_1 = i] \mathbb{P}(L_1 = i)$$
$$= \sum_{i=1}^{N_R} p_R(X_i) \int_S \eta_i([X], y) y y^T \, dy \mathbb{P}(L_1 = i). \quad \text{(D14)}$$

Next,

$$\mathbb{E}[(G_1 Y_1 - \langle G_1 Y_1 \rangle)(G_2 Y_2 - \langle G_2 Y_2 \rangle)^T] = \mathbb{E}[(G_1 Y_1)(G_2 Y_2)^T] - \mathbb{E}[(G_1 Y_1)] \mathbb{E}[(G_2 Y_2)^T]. \quad \text{(D15)}$$

and

$$\mathbb{E}[(G_1 Y_1)(G_2 Y_2)^T] = \sum_{m=1}^{N_R} \sum_{i=1}^{N_R} \mathbb{E}[(G_1 Y_1)(G_2 Y_2)^T \mid L_1 = i, \, L_2 = m] \mathbb{P}(L_1 = i, L_2 = m)$$
$$= \sum_{m=1}^{N_R} \sum_{i=1}^{N_R} \mathbb{E}[R_i R_m] \mathbb{E}[Y_1 Y_2^T \mid L_1 = i, \, L_2 = m] \mathbb{P}(L_1 = i) \mathbb{P}(L_2 = m).$$
$$\text{(D16)}$$

bioRxiv preprint doi: https://doi.org/10.1101/2025.04.18.649595; this version posted February 18, 2026. The copyright holder for this preprint (which was not certified by peer review) is the author/funder, who has granted bioRxiv a license to display the preprint in perpetuity. It is made available under aCC-BY 4.0 International license.

The case $i \neq m$ means that the two G proteins are linked to a different receptor (whose states are independent). In this case, $\mathbb{E}[R_i R_m] = \mathbb{E}[R_i]\mathbb{E}[R_m] = p_R(X_i)p_R(X_m)$. However, the case $i = m$ means that both G proteins are linked to the same receptor. By the calculation above (with $n = 2$), we have

$$\mathbb{E}[R_i(t_j)R_i(t_\ell)] = p_R(X_i)^2 + \frac{p_R(X_i)(1-p_R(X_i))}{1+\lambda(X_i)\tau}.$$

Therefore,

$$\begin{aligned}\mathbb{E}[(G_1Y_1)(G_2Y_2)^T] &= \sum_{i=1}^{N_R}\left(p_R(X_i)^2 + \frac{p_R(X_i)(1-p_R(X_i))}{1+\lambda(X_i)\tau}\right)\mathbb{E}[Y_1Y_2^T \mid L_1 = L_2 = i]\mathbb{P}(L_1 = L_2 = i)\\ &+ 2\sum_{i<m}^{N_R} p_R(X_i)p_R(X_m)\mathbb{E}[Y_1Y_2^T \mid L_1 = i,\, L_2 = m]\mathbb{P}(L_1 = i)\mathbb{P}(L_2 = m).\end{aligned} \tag{D17}$$

Putting this all together, we obtain

$$\mathbb{E}[(G_1Y_1 - \langle G_1Y_1\rangle)(G_2Y_2 - \langle G_2Y_2\rangle)^T] = \sum_{i=1}^{N_R}\left(\frac{p_R(X_i)(1-p_R(X_i))}{1+\lambda(X_i)\tau}\right)\mathbb{E}[Y_1Y_2^T \mid L_1 = L_2 = i]\mathbb{P}(L_1 = L_2 = i) \tag{D18}$$

and the covariance matrix $\Sigma_G = \mathbb{E}[(V_G - \langle V_G\rangle)(V_G - \langle V_G\rangle)^T]$ is:

$$\begin{aligned}\Sigma_G &= N_G\sum_{i=1}^{N_R} p_R(X_i)\int_S \eta_i([X],y)yy^T\,dy\mathbb{P}(L_1 = i)\\ &- N_G\sum_{m=1}^{N_R}\sum_{i=1}^{N_R} p_R(X_m)p_R(X_i)\int_S\int_S \eta_m([X],z)\eta_i([X],y)yz^T\,dy\,dz\mathbb{P}(L_1 = i)\mathbb{P}(L_2 = m)\\ &+ (N_G^2 - N_G)\sum_{i=1}^{N_R}\left(\frac{p_R(X_i)(1-p_R(X_i))}{1+\lambda(X_i)\tau}\right)\mathbb{E}[Y_1Y_2^T \mid L_1 = L_2 = i]\mathbb{P}(L_1 = L_2 = i).\end{aligned} \tag{D19}$$

By definition of η_i,

$$\mathbb{E}[Y_1Y_2^T \mid L_1 = L_2 = i] = \int_S\int_S \eta_i([X],z)\eta_i([X],y)yz^T\,dy\,dz.$$

5. Derivation of Proposition D.2

For any continuous function f on the sphere S, when N_R is large enough we have

$$\frac{1}{N_R}\sum_{i=1}^{N_R} f(X_i) \approx \frac{1}{|S|}\int_S f(x)\,dx, \tag{D20}$$

bioRxiv preprint doi: https://doi.org/10.1101/2025.04.18.649595; this version posted February 18, 2026. The copyright holder for this preprint (which was not certified by peer review) is the author/funder, who has granted bioRxiv a license to display the preprint in perpetuity. It is made available under aCC-BY 4.0 International license.

with high probability. This is justified by the law of large numbers, since the receptor positions $\{X_i\}$ are independent. Using this and the approximations (D7) and (D8), we have

$$\begin{aligned}\mu_G &= N_G \sum_{i=1}^{N_R} p_R(X_i) \int_S \eta_i([X], y) y \, dy \mathbb{P}(L_1 = i) \\ &\approx N_G \left(\frac{1}{N_R} \sum_{i=1}^{N_R} p_R(X_i) X_i \right) \approx N_G \frac{1}{|S|} \int_S p_R(x) x \, dx, \end{aligned} \tag{D21}$$

and similarly,

$$\begin{aligned} M_1 &\approx \frac{1}{N_R} \sum_{i=1}^{N_R} p_R(X_i) X_i X_i^T - \left(\frac{1}{N_R} \sum_{i=1}^{N_R} p_R(X_i) X_i \right) \left(\frac{1}{N_R} \sum_{m=1}^{N_R} p_R(X_m) X_m \right) \\ &\approx \frac{1}{|S|} \int_S p_R(x) x x^T \, dx - \left(\frac{1}{|S|} \int_S p_R(x) x \, dx \right) \left(\frac{1}{|S|} \int_S p_R(y) y^T \, dy \right). \end{aligned} \tag{D22}$$

Since L_1 and L_2 are independent, $\mathbb{P}(L_1 = L_2 = i) = \mathbb{P}(L_1 = i)\mathbb{P}(L_2 = i) \approx (N_R)^{-2}$. Therefore, using the same reasoning as for M_1, we have

$$M_2 \approx \frac{1}{N_R} \sum_{i=1}^{N_R} \frac{p_R(X_i)(1 - p_R(X_i))}{1 + \tau\lambda(X_i)} X_i X_i^T \approx \frac{1}{|S|} \int_S \frac{p_R(x)(1 - p_R(x))}{1 + \tau\lambda(x)} x x^T \, dx.$$

Appendix E: Local concentration noise versus direction noise

Here is a simple illustration of the phenomenon that in the ratiometric model the gradient of active G protein may be stronger than in the classical model even while the local noise in active G protein may be greater for the ratiometric model. Thus, high-variability in local concentration of active G protein is not necessarily an indicator of poor direction estimation. This is because direction estimation involves a comparison across multiple different regions of the cell surface.

Imagine a greatly simplified, one-dimensional model where the cell has a front and a back: at the front there are N_R receptors and at the back there are N_R receptors. Let $\epsilon \in (0, 1/2)$ be a small parameter representing a small gradient of ligand concentration (toward the front end). Let us suppose that R_f^+ and R_b^+ are independent random variables with distributions

$$R_f^+ \sim \text{Binomial}\left(N_R, \frac{1}{2} + \epsilon\right), \qquad R_b^+ \sim \text{Binomial}\left(N_R, \frac{1}{2} - \epsilon\right).$$

These R_f^+ and R_b^+ represent the number of active receptors at the front and back of the cell, respectively. This statistical model is equivalent to saying that the N_R proteins at the front

bioRxiv preprint doi: https://doi.org/10.1101/2025.04.18.649595; this version posted February 18, 2026. The copyright holder for this preprint (which was not certified by peer review) is the author/funder, who has granted bioRxiv a license to display the preprint in perpetuity. It is made available under aCC-BY 4.0 International license.

end have independent states, each being active with probability $\frac{1}{2}+\epsilon$. Similarly, at the back end, the probability of a receptor being active is $\frac{1}{2}-\epsilon$. Then define

$$R_f^- = N_R - R_f^+, \qquad R_b^- = N_R - R_b^+,$$

which represent the number of inactive receptor at the front and back of the cell, respectively. The mean receptor levels are

$$\mathbb{E}[R_f^\pm] = N_R\left(\frac{1}{2}\pm\epsilon\right), \qquad \mathbb{E}[R_b^\pm] = N_R\left(\frac{1}{2}\mp\epsilon\right)$$

and the variances are

$$\mathrm{Var}(R_f^+) = \mathrm{Var}(R_f^-) = \mathrm{Var}(R_b^+) = \mathrm{Var}(R_b^-) = N_R\left(\frac{1}{2}+\epsilon\right)\left(\frac{1}{2}-\epsilon\right).$$

So, with $\epsilon > 0$, the receptor gradient has a small bias toward the front end:

$$\mathbb{E}[R_f^+ - R_b^+] = 2N_R\epsilon > 0.$$

In addition to receptor, there are G proteins at the front and back. Suppose there are N_G proteins at the front and N_G proteins at the back. Random variables G_f^+ and G_b^+ will be the number of active G proteins at the front and back, respectively. Given R_f^+ and R_f^-, G proteins at the front end will be active with probability that depends on R_f^+ and R_f^-; what distinguishes the ratiometric from classical models is how this probability depends on R_f^+ and R_f^-. In the ratiometric model, the steady-state probability that a G protein at the front end is active is

$$\frac{R_f^+}{R_f^+ + R_f^-} = \frac{R_f^+}{N_R}.$$

(Rate constants are normalized to one.) In the classical model, the inactivation rate is taken to be constant and uniform across the cell (same at the front and back). Therefore, in the classical model, the steady state probability that a G protein at the front end is active has the form

$$\frac{R_f^+/N_R}{R_f^+/N_R + c}$$

for some constant $c > 0$. Similar statements hold at the back end of the cell, with R_b^+ replacing R_f^+. In either case, classical or ratiometic, the probability that a G protein is active, is a function of the local fraction of active receptor.

bioRxiv preprint doi: https://doi.org/10.1101/2025.04.18.649595; this version posted February 18, 2026. The copyright holder for this preprint (which was not certified by peer review) is the author/funder, who has granted bioRxiv a license to display the preprint in perpetuity. It is made available under aCC-BY 4.0 International license.

In view of this, we take G_f^+ and G_b^+ to be independent random variables having the following distribution: given R_f^+ and R_b^+,

$$G_f^+ \sim \text{Binomial}\left(N_G, f\left(\frac{R_f^+}{N_R}\right)\right), \qquad G_b^+ \sim \text{Binomial}\left(N_G, f\left(\frac{R_b^+}{N_R}\right)\right).$$

where

$$f(r) = \begin{cases} r, & \text{for the ratiometric model,} \\ \frac{r}{r+c}, & \text{for the classical model.} \end{cases}$$

This statistical model is equivalent to saying that the N_G proteins at the front end have independent states, each being active with probability $f\left(\frac{R_f^+}{N_R}\right)$. And similarly for the proteins at the back end.

To compare the two models, we choose the constant c so that the mean total activation rate of G protein is the same. The mean total activation is

$$\mathbb{E}[G_f^+ + G_b^+] = N_G\mathbb{E}[f(R_f^+/N_R)] + N_G\mathbb{E}[f(R_b^+/N_R)].$$

For the ratiometric model, the function f is linear ($f(r) = r$), so this can be computed explicitly:

$$\mathbb{E}[G_f^+ + G_b^+] = N_G\mathbb{E}[R_f^+/N_R] + N_G\mathbb{E}[R_b^+/N_R] = N_G.$$

Therefore, the matching condition requires that the constant c (in the classical model) be chosen so that

$$\mathbb{E}\left[\frac{R_f^+/N_R}{R_f^+/N_R + c}\right] + \mathbb{E}\left[\frac{R_b^+/N_R}{R_b^+/N_R + c}\right] = 1. \qquad \text{(E1)}$$

This relation for c cannot be solved explicitly. Nevertheless, we claim that when N_R is large and ϵ is small, $c \approx 1/2$. To see this, note that when N_R is large, the distributions of R_f/N_R and R_b/N_R are very concentrated around $\frac{1}{2} + \epsilon$ and $\frac{1}{2} - \epsilon$, respectively. Therefore, when $\epsilon > 0$ is small, (E1) implies that

$$\frac{\frac{1}{2} + \epsilon}{\frac{1}{2} + \epsilon + c} + \frac{\frac{1}{2} - \epsilon}{\frac{1}{2} - \epsilon + c} \approx 1,$$

which implies that $c \approx 1/2$ for small ϵ.

In the case of the classical model, f is nonlinear, and this makes it impossible to calculate explicitly the mean and variance of G_f^+ and G_b^+ in that case. However, as we explained above,

bioRxiv preprint doi: https://doi.org/10.1101/2025.04.18.649595; this version posted February 18, 2026. The copyright holder for this preprint (which was not certified by peer review) is the author/funder, who has granted bioRxiv a license to display the preprint in perpetuity. It is made available under aCC-BY 4.0 International license.

when N_R is large and ϵ is small, the constant c is approximately $1/2$, and the function f may be approximated well by its linear expansion at the value $r = 1/2$:

$$\frac{r}{r+1/2} \approx \frac{1}{2} + \frac{1}{2}\left(r - \frac{1}{2}\right)$$

This motivates the following modification, which makes calculation explicit in both models: Given R_f^+ and R_b^+, let

$$G_f^+ \sim \text{Binomial}\left(N_G, \ell\left(\frac{R_f^+}{N_R}\right)\right), \qquad G_b^+ \sim \text{Binomial}\left(N_G, \ell\left(\frac{R_b^+}{N_R}\right)\right).$$

where, instead of f defined above, we use the function

$$\ell(r) = \begin{cases} r, & \text{for the ratiometric model,} \\ \frac{1}{2} + \frac{1}{2}\left(r - \frac{1}{2}\right), & \text{for the classical model.} \end{cases} \tag{E2}$$

This ℓ is linear in both cases, and it has the form

$$\ell(r) = \frac{1}{2} + \alpha\left(r - \frac{1}{2}\right), \quad \alpha = \begin{cases} 1, & \text{for the ratiometric model,} \\ \frac{1}{2}, & \text{for the classical model.} \end{cases}$$

With this modification, the means and variances of G_f^+ and G_b^+ are computable in both models. We compute:

$$\mathbb{E}[G_f^+] = N_G \mathbb{E}\left[\ell\left(\frac{R_f^+}{N_R}\right)\right] = N_G\left(\frac{1}{2} + \alpha\left(\mathbb{E}[R_f^+/N_R] - \frac{1}{2}\right)\right) = N_G\left(\frac{1}{2} + \alpha\epsilon\right), \tag{E3}$$

$$\mathbb{E}[G_b^+] = N_G \mathbb{E}\left[\ell\left(\frac{R_b^+}{N_R}\right)\right] = N_G\left(\frac{1}{2} + \alpha\left(\mathbb{E}[R_b^+/N_R] - \frac{1}{2}\right)\right) = N_G\left(\frac{1}{2} - \alpha\epsilon\right). \tag{E4}$$

In particular, the total activation level is the same for all α:

$$\mathbb{E}[G_f^+ + G_b^+] = N_G, \tag{E5}$$

while the mean front-to-back gradient in active G protein is

$$\mathbb{E}[G_f^+ - G_b^+] = 2N_G\alpha\epsilon = \alpha(N_G/N_R)\mathbb{E}[R_f^+ - R_b^+]. \tag{E6}$$

Recall that the ratiometric case corresponds exactly to $\alpha = 1$, while the classical case corresponds to $\alpha = 1/2$, so the mean gradient is twice as large in the ratiometric case.

bioRxiv preprint doi: https://doi.org/10.1101/2025.04.18.649595; this version posted February 18, 2026. The copyright holder for this preprint (which was not certified by peer review) is the author/funder, who has granted bioRxiv a license to display the preprint in perpetuity. It is made available under aCC-BY 4.0 International license.

Now, let us consider how the variances depend on α, especially the cases $\alpha = 1$ (ratiometric) and $\alpha = 1/2$ (classical). The variance in active G protein, at both the front and back end, can be computed explicitly:

$$\mathrm{Var}(G_f^+) = N_G \mathbb{E}\left[\ell\left(\frac{R_f^+}{N_R}\right)\right]\left(1 - \mathbb{E}\left[\ell\left(\frac{R_f^+}{N_R}\right)\right]\right) + (N_G^2 - N_G)\mathrm{Var}\left(\ell\left(\frac{R_f^+}{N_R}\right)\right) \tag{E7}$$

$$= N_G\left(\frac{1}{2} + \alpha\epsilon\right)\left(\frac{1}{2} - \alpha\epsilon\right) + (N_G^2 - N_G)\frac{\alpha^2}{N_R}\left(\frac{1}{2} + \alpha\epsilon\right)\left(\frac{1}{2} - \alpha\epsilon\right). \tag{E8}$$

So, the coefficient of variation of G_f^+ is

$$\mathrm{CV}^2(G_f^+) = \frac{\mathrm{Var}(G_f^+)}{\mathbb{E}[G_f^+]^2} = \frac{N_G\left(\frac{1}{2} + \alpha\epsilon\right)\left(\frac{1}{2} - \alpha\epsilon\right) + (N_G^2 - N_G)\frac{\alpha^2}{N_R}\left(\frac{1}{2} + \alpha\epsilon\right)\left(\frac{1}{2} - \alpha\epsilon\right)}{\left(N_G\left(\frac{1}{2} + \alpha\epsilon\right)\right)^2} \tag{E9}$$

$$= \left(\frac{1}{N_G} + \left(1 - \frac{1}{N_G}\right)\frac{\alpha^2}{N_R}\right)\frac{\left(\frac{1}{2} - \alpha\epsilon\right)}{\left(\frac{1}{2} + \alpha\epsilon\right)}. \tag{E10}$$

A similar compuation shows that at the back end we have

$$\mathrm{CV}^2(G_b^+) = \left(\frac{1}{N_G} + \left(1 - \frac{1}{N_G}\right)\frac{\alpha^2}{N_R}\right)\frac{\left(\frac{1}{2} + \alpha\epsilon\right)}{\left(\frac{1}{2} - \alpha\epsilon\right)}. \tag{E11}$$

For small ϵ, this is approximately

$$\mathrm{CV}^2(G_f^+) \approx \frac{1}{N_G} + \left(1 - \frac{1}{N_G}\right)\frac{\alpha^2}{N_R}, \qquad \mathrm{CV}^2(G_b^+) \approx \frac{1}{N_G} + \left(1 - \frac{1}{N_G}\right)\frac{\alpha^2}{N_R}.$$

In particular, these quantities increase with α for $\alpha \in [0, 1]$. On the other hand, the mean front-to-back gradient

$$\mathbb{E}[G_f^+ - G_b^+] = 2N_G\alpha\epsilon$$

also increases with α. This shows that the directional signal gets stronger with larger α, even though the local noise levels (measured by coefficients of variation $\mathrm{CV}^2(G_f^+)$ and $\mathrm{CV}^2(G_b^+)$) are larger with larger α. In particular, this illustrates the phenomenon that with the ratiometric model ($\alpha = 1$), the local noise in G_f^+ and G_b^+ may be greater than in the classical model ($\alpha = 1/2$), even while the front-to-back gradient in G protein is stronger for the ratiometric model.

This phenomenon can be seen more clearly from examining the joint distributions of $(R_f^+/N_R, G_f^+/N_G)$ and $(R_b^+/N_R, G_b^+/N_G)$ for the two models, see Figure 11. In Figure 12, we plot histrograms representing the marginal distributions of G_f^+/N_G and G_b^+/N_G for the two models. The marginal distributions of G_f^+/N_G and G_b^+/N_G have higher variance for the

bioRxiv preprint doi: https://doi.org/10.1101/2025.04.18.649595; this version posted February 18, 2026. The copyright holder for this preprint (which was not certified by peer review) is the author/funder, who has granted bioRxiv a license to display the preprint in perpetuity. It is made available under aCC-BY 4.0 International license.

ratiometric model, than for the classical model. Figure 13 shows a histrogram representing the distribution of active G protein gradient $(G_f^+ - G_b^+)/N_G$ for both models. We observe that the distribution of this gradient is biased to the right (front end) for the ratiometric model, as explained in the analysis above. From Figure 11, we see how the different activation functions for the ratiometric vs. classical models leads to a bias in the G protein gradient from front to back, producing stronger (steeper) gradient in the ratiometric case, even while the variance in local G protein activation is higher with the ratiometric model.

Appendix F: Classical model

Here we explain why the classical model (nonratiometric) performs more poorly than the ratiometric model. Recall that V_G^r and V_G^c refer to the vector (B4) under the ratiometric and classical models, respectively. We have seen that the ratiometric model V_G^r performs particularly well compared to V_{RL} and V_C when the time scales are such that the G proteins are approximately independent, and $N_G > N_R$. In principle, the classical model estimate V_G^c might also perform well in this scenario, yet our simulations show that this is not the case. An important point derived above is that for the ratiometric model, with N_R sufficiently large, the mean resultant vector is

$$\mathbb{E}[V_G^r] = \frac{N_G}{|S|} \int_S p_G^r(y) y \, dy \approx \frac{N_G}{|S|} \int_S p_R(y) y \, dy$$

where $p_G^r(y)$ is the probability that a G protein at location y is active (under the ratiometric model); $p_R(y)$ is the probability that a receptor at y is active. In particular, the mean vector $\mathbb{E}[V_G^r]$ does not depend on $\lambda_0 \tau_{\text{dif}}$, where $\tau_{\text{dif}} = \tau$ is the diffusive encounter time scale from (D1), and λ_0^{-1} is the time scale of receptor switching. For the classical model, there is an additional time scale $\tau_{\text{off}} = 1/k_i'$, where k_i' is the rate at which G proteins deactivate randomly in the classical model. For the classical model, the mean vector $\mathbb{E}[V_G^c]$ depends on all of these three time scales τ_{off}, τ_{dif}, and λ_0^{-1} in a way that makes the classical model less effective at direction estimation than the ratiometric model.

For the classical model, let $p_G^c(y)$ denote the probability that a G protein at position y is active. The probability p_G^c cannot be computed explicitly, but we analyze an approximate model that has similar features and gives insight into the way that p_G^c depends on the time scales. As we will show, the gradient of p_G^c may be much flatter than that of p_R or p_G^r. In

bioRxiv preprint doi: https://doi.org/10.1101/2025.04.18.649595; this version posted February 18, 2026. The copyright holder for this preprint (which was not certified by peer review) is the author/funder, who has granted bioRxiv a license to display the preprint in perpetuity. It is made available under aCC-BY 4.0 International license.

the classical model, the state $G(t)$ of a G protein at time t and position $Y(t) = y$ is active if and only if that G protein encountered an active receptor (possibly multiple) at some time between time $t - T$ and t, where the random time T has the Exponential distribution with mean τ_{off}. Otherwise, that protein is inactive. The number of receptors that a G protein encounters before deactivation is random and it depends in a complicated way on the receptor configuration, but it is reasonable to suppose that random number N of distinct encounters has mean $\mathbb{E}[N] = \tau_{\text{off}}/\tau_{\text{dif}}$, where

$$\tau_{\text{dif}} = \frac{L^2}{N_R D_G} \ln(\delta/r_*)$$

is the diffusive encounter time scale (see (D1)). Indeed, looking backward in time we may assume that the encounters between a G protein and receptors occur as a Poisson arrival process at rate $1/\tau_{\text{dif}}$ (times between encounters are approximately exponentially distributed with mean τ_{dif}). Thus, N has the distribution of A_T, where A_t is such a Poisson arrival process and $T \sim \text{Exponential}(1/\tau_{\text{off}})$ is independent of the process A. This implies that the number N of distinct receptor encounters has the (shifted) Geometric distribution:

$$\begin{aligned} \mathbb{P}(N = n) &= \int_0^\infty \mathbb{P}(A_t = n \mid T = t) \frac{1}{\tau_{\text{off}}} e^{-t/\tau_{\text{off}}}\, dt \\ &= \left(\frac{\tau_{\text{dif}}}{\tau_{\text{dif}} + \tau_{\text{off}}}\right) \left(\frac{\tau_{\text{off}}}{\tau_{\text{dif}} + \tau_{\text{off}}}\right)^n, \qquad n = 0, 1, 2, 3, \ldots \end{aligned} \tag{F1}$$

with mean $\mathbb{E}[N] = \tau_{\text{off}}/\tau_{\text{dif}}$. This distribution is in close agreement with our simulations; see Figure 14 where we show numerical computation of the empirical distribution of distinct receptors encountered by a G protein before its state resets (in the classical model).

An important consequence of this is that the probability that a G protein encounters zero receptors between resetting events is:

$$\mathbb{P}(N = 0) = \frac{1}{1 + \tau_{\text{off}}/\tau_{\text{dif}}} = \frac{1}{1 + \mathbb{E}[N]}.$$

Such G proteins carry zero information about the receptor states, and thus zero information about the pheromone gradient or local concentration. This is an important difference between the classical and ratiometric models: in the classical model, at a given time t, some fraction (approximately $\frac{1}{1+\tau_{\text{off}}/\tau_{\text{dif}}}$) of proteins carry zero information about the local pheromone concentration because they have not encountered any receptor (active or inactive) since the most recent state resetting event; in the ratiometric model, however, every G

bioRxiv preprint doi: https://doi.org/10.1101/2025.04.18.649595; this version posted February 18, 2026. The copyright holder for this preprint (which was not certified by peer review) is the author/funder, who has granted bioRxiv a license to display the preprint in perpetuity. It is made available under aCC-BY 4.0 International license.

protein carries some information about the local pheromone concentration, via the state of the most recently encountered receptor. Note that $\mathbb{P}(N = 0)$ does not depend in any way on the pheromone concentration; $\mathbb{E}[N]$ depends on the density of receptors, but not on their states.

The presence of "uninformative" G proteins (in the classical model) carrying no information about the pheromone gradient leads to what we call a flattening of the gradient of p_G^c relative to p_R. Assuming encounters are nearly instantaneous, the probability that a given encounter at location x activates the G protein is approximately $p_R(x)$. Assuming that the N encounters are local, then the probability that a G protein at x (under classical model) will be active is then

$$\begin{aligned} p_G^c(x) &= 1 - \mathbb{E}[(1 - p_R(x))^N] && \text{(F2)} \\ &= 1 - \sum_{k=0}^{\infty} (1 - p_R(x))^k q(1-q)^k, \qquad q = \frac{\tau_{\text{dif}}}{\tau_{\text{dif}} + \tau_{\text{off}}} && \text{(F3)} \\ &= \frac{p_R(x)(1-q)}{q + p_R(x)(1-q)} && \text{(F4)} \\ &= \frac{p_R(x)\mathbb{E}[N]}{1 + p_R(x)\mathbb{E}[N]}. && \text{(F5)} \end{aligned}$$

Thus the relationship between p_G^c and p_R depends on the mean number of receptor encounters $\mathbb{E}[N]$. In Figure 15 we plot p_G^c versus p_R for different values of $\mathbb{E}[N]$. When $\tau_{\text{off}}/\tau_{\text{diff}} = \mathbb{E}[N] <$ < 1, most G proteins will be inactive, and (F5) shows that

$$\mathbb{E}[N] \ll 1 \qquad \Longrightarrow \qquad p_G^c(x) \approx 0, \quad \text{for all } \ x. \tag{F6}$$

However, when $\tau_{\text{off}}/\tau_{\text{diff}} = \mathbb{E}[N] \gg 1$ and p_R not too small, there is saturation – most G proteins will be active, and (F5) shows that

$$\mathbb{E}[N] \gg 1 \qquad \Longrightarrow \qquad p_G^c(x) \approx 1, \quad \text{for all } \ x. \tag{F7}$$

The gradient of p_G^c is

$$\nabla p_G^c(x) = \frac{\mathbb{E}[N]}{(1 + p_R(x)\mathbb{E}[N])^2} \nabla p_R(x). \tag{F8}$$

The prefactor in front of ∇p_R can be larger or smaller than 1. We have

$$\frac{\mathbb{E}[N]}{(1 + p_R(x)\mathbb{E}[N])^2} \geq 1$$

bioRxiv preprint doi: https://doi.org/10.1101/2025.04.18.649595; this version posted February 18, 2026. The copyright holder for this preprint (which was not certified by peer review) is the author/funder, who has granted bioRxiv a license to display the preprint in perpetuity. It is made available under aCC-BY 4.0 International license.

if only if $p_R(x) < \frac{1}{\sqrt{\mathbb{E}[N]}} - \frac{1}{\mathbb{E}[N]}$. In particular, if $p_R(x) > \frac{1}{\sqrt{\mathbb{E}[N]}} - \frac{1}{\mathbb{E}[N]}$, then there will necessarily be a flattening effect whereby the prefactor in (F8) is less than 1. Our earlier analysis shows that for shallow gradients, the NSR is smallest when $C_0 \approx K_d$, which implies p_R is near 1/2. In this regime, p_G^c will be flatter than p_R, as shown in Figure 15. In particular, p_G^c is flatter than p_G^r, since for the ratiometric model $p_G^r \approx p_R$ regardless of the time scales. The flattening of p_G^c versus p_R and p_G^r implies less accuracy (higher NSR) for the classical method, since the NSR scales inversely with the gradient squared (i.e. as $1/b^2$ in (B12).)

Appendix G: Captions for Movies

Movie S1. Temporal dynamics of receptor and G-protein vector orientations during Classical gradient sensing. **Top left:** Full simulation showing all receptor and G-protein positions; receptors are color-coded by activation state. **Top middle:** Vector orientations for active (yellow), inactive (gray), and combined (blue) receptor populations. **Top right:** Vectors for the combined receptor (blue) and G-protein (green) populations. The black horizontal line denotes the true gradient direction. **Bottom:** Time traces showing the angle between the pheromone gradient and the receptor (green) and G-protein (cyan) vectors. NG = 2500, NR = 375, D_m = 0.002 $\mu m^2/s$, k_{gi} = 0.0083 s^{-1}, gradient = 8 to 4 nM. `https://youtu.be/beVWFQ6Jzdg`

Movie S2. Temporal dynamics of receptor and G-protein vector orientations during Classical gradient sensing. **Top left:** Full simulation showing all receptor and G-protein positions; receptors are color-coded by activation state. **Top middle:** Vector orientations for active (yellow), inactive (gray), and combined (blue) receptor populations. **Top right:** Vectors for the combined receptor (blue) and G-protein (green) populations. The black horizontal line denotes the true gradient direction. **Bottom:** Time traces showing the angle between the pheromone gradient and the receptor (green) and G-protein (cyan) vectors. NG = 2500, NR = 1500, D_m = 0.002 $\mu m^2/s$, k_{gi} = 0.0357 s^{-1}, gradient = 8 to 4 nM. `https://youtu.be/nvEN7Sysycc`

Movie S3. Temporal dynamics of receptor and G-protein vector orientations during Classical gradient sensing. **Top left:** Full simulation showing all receptor and G-protein positions; receptors are color-coded by activation state. **Top middle:** Vector orientations for active (yellow), inactive (gray), and combined (blue) receptor populations. **Top right:**

bioRxiv preprint doi: https://doi.org/10.1101/2025.04.18.649595; this version posted February 18, 2026. The copyright holder for this preprint (which was not certified by peer review) is the author/funder, who has granted bioRxiv a license to display the preprint in perpetuity. It is made available under aCC-BY 4.0 International license.

Vectors for the combined receptor (blue) and G-protein (green) populations. The black horizontal line denotes the true gradient direction. **Bottom:** Time traces showing the angle between the pheromone gradient and the receptor (green) and G-protein (cyan) vectors. NG = 2500, NR = 6000, D_m = 0.002 $\mu m^2/s$, k_{gi} = 0.1687 s^{-1}, gradient = 8 to 4 nM. `https://youtu.be/egZNPnUeXfM`

Movie S4. Temporal dynamics of receptor and G-protein vector orientations during Ratiometric gradient sensing. **Top left:** Full simulation showing all receptor and G-protein positions; receptors are color-coded by activation state. **Top middle:** Vector orientations for active (yellow), inactive (gray), and combined (blue) receptor populations. **Top right:** Vectors for the combined receptor (blue) and G-protein (green) populations. The black horizontal line denotes the true gradient direction. **Bottom:** Time traces showing the angle between the pheromone gradient and the receptor (green) and G-protein (cyan) vectors. NG = 2500, NR = 375, D_m = 0.002 $\mu m^2/s$, gradient = 8 to 4 nM. `https://youtu.be/B3Dg8SMIhEE`

Movie S5. Temporal dynamics of receptor and G-protein vector orientations during Ratiometric gradient sensing. **Top left:** Full simulation showing all receptor and G-protein positions; receptors are color-coded by activation state. **Top middle:** Vector orientations for active (yellow), inactive (gray), and combined (blue) receptor populations. **Top right:** Vectors for the combined receptor (blue) and G-protein (green) populations. The black horizontal line denotes the true gradient direction. **Bottom:** Time traces showing the angle between the pheromone gradient and the receptor (green) and G-protein (cyan) vectors. NG = 2500, NR = 1500, D_m = 0.002 $\mu m^2/s$, gradient = 8 to 4 nM. `https://youtu.be/9SNJQRN9yAQ`

Movie S6. Temporal dynamics of receptor and G-protein vector orientations during Ratiometric gradient sensing. **Top left:** Full simulation showing all receptor and G-protein positions; receptors are color-coded by activation state. **Top middle:** Vector orientations for active (yellow), inactive (gray), and combined (blue) receptor populations. **Top right:** Vectors for the combined receptor (blue) and G-protein (green) populations. The black horizontal line denotes the true gradient direction. **Bottom:** Time traces showing the angle between the pheromone gradient and the receptor (green) and G-protein (cyan) vectors. NG = 2500, NR = 6000, D_m = 0.002 $\mu m^2/s$, gradient = 8 to 4 nM. `https://youtu.be/`

bioRxiv preprint doi: https://doi.org/10.1101/2025.04.18.649595; this version posted February 18, 2026. The copyright holder for this preprint (which was not certified by peer review) is the author/funder, who has granted bioRxiv a license to display the preprint in perpetuity. It is made available under aCC-BY 4.0 International license.

JxBwMO2otD8
